# Imidazole-Functionalized Thieno[3,2-c]Quinoline Hybrids in Aggressive Medullary Thyroid Cancer Cell Models: Biological Evaluation and in Silico Insights

**DOI:** 10.3390/ph19071037

**Published:** 2026-07-03

**Authors:** Gabriele La Monica, Alessia Bono, Federica Alamia, Dennis Tocco, Giuseppe Pizzolanti, Antonino Lauria, Annamaria Martorana

**Affiliations:** 1 Dipartimento di Scienze e Tecnologie Biologiche Chimiche e Farmaceutiche “STEBICEF”, University of Palermo, 90123 Palermo, Italy; alessia.bono01@unipa.it (A.B.); federica.alamia01@unipa.it (F.A.); dennis.tocco@unipa.it (D.T.); antonino.lauria@unipa.it (A.L.); annamaria.martorana@unipa.it (A.M.); 2 Fondazione Umberto Veronesi (FUV), Via Solferino 19, 20121 Milano, Italy; 3 Department of Health Promotion, Mother and Child Care, Internal Medicine and Medical Specialties (PROMISE), University of Palermo, 90127 Palermo, Italy; giuseppe.pizzolanti@unipa.it; 4 Advanced Technologies Network Center (ATEN Center), University of Palermo, 90128 Palermo, Italy

**Keywords:** medullary thyroid cancer, thienoquinoline, in silico analysis, antiproliferative activity, TT, MZ-CRC-1, imidazole hybrids, molecular docking, molecular dynamics simulations

## Abstract

**Background/Objectives**: Medullary thyroid carcinoma (MTC) is a rare and aggressive endocrine malignancy frequently associated with RET alterations and dysregulation of RET-associated signaling pathways. In previous studies, first-generation nitro-substituted thieno[3,2-c]quinolines **1** showed promising antiproliferative activity in TT(RET^C634R^) cells, while subsequent imidazole-based optimization generated second-generation derivatives **2** with broad-spectrum antiproliferative activity in the NCI-60 human tumor cell line panel. Since thyroid cancer models are not included in the NCI-60 platform, the present study aimed to evaluate the antiproliferative potential of these optimized derivatives in clinically relevant MTC cellular models. **Methods**: Imidazole-functionalized thieno[3,2-c]quinoline derivatives (**2a**–**j**) were evaluated against TT(RET^C634R^) and MZ-CRC-1(RET^M918T^) cells. The most active compounds were further characterized through induced fit docking (IFD), MM-GBSA calculations, and molecular dynamics (MD) simulations on representative molecular targets. In silico ADME/Toxicity profiling was also performed to assess their developability. **Results**: Several derivatives exhibited potent activity in both MTC cell models, with multiple compounds achieving submicromolar potency. Compounds **2b** and **2g** emerged as the most active derivatives in TT(RET^C634R^) cells, whereas compounds **2d** and **2i** displayed the most favorable profile in the aggressive MZ-CRC-1(RET^M918T^) model, thereby extending the biological applicability of the scaffold to an additional clinically relevant RET-mutant context. Computational studies supported RET as the most plausible molecular target for all selected lead compounds, while favorable PI3Kα interaction profiles were predicted for selected derivatives, suggesting distinct target engagement profiles within the series. The most promising derivatives also exhibited an overall favorable predicted ADME/Toxicity profile. **Conclusions**: The present findings support the success of imidazole-based optimization of the thieno[3,2-c]quinoline scaffold and identify thieno[3,2-c]quinolines **2** as promising lead structures for the future development of novel anti-MTC agents. Further biological and mechanistic investigations will be necessary to better clarify the molecular mechanisms underlying their antiproliferative activity and to guide future lead optimization studies.

## 1. Introduction

Thyroid cancer (TC) is one of the most common endocrine malignancies and ranks among the ten most frequently diagnosed cancers worldwide, with a steadily increasing incidence, particularly in women [1,2,3]. Based on histopathological features, TC is classified into differentiated thyroid cancer (DTC), anaplastic thyroid cancer (ATC), and medullary thyroid cancer (MTC). MTC is a neuroendocrine tumor arising from parafollicular C cells that accounts for approximately 5% of thyroid malignancies but more than 15% of thyroid cancer-related deaths because of its aggressive behaviour, early metastatic spread, high recurrence rate, and limited responsiveness to conventional therapies [1,2,4,5,6]. Approximately 75% of cases are sporadic, whereas the remaining 25% are hereditary and mainly associated with familial medullary thyroid cancer (FMTC) and Multiple Endocrine Neoplasia type 2 (MEN2A and MEN2B) syndromes [4,5].

From a molecular perspective, the pathogenesis of medullary thyroid carcinoma is mainly driven by activating mutations and functional alterations affecting specific signaling pathways and regulatory proteins. Among these, the rearranged during transfection (RET) proto-oncogene represents one of the major molecular drivers in both hereditary and sporadic MTC. RET encodes a receptor tyrosine kinase involved in the regulation of cell proliferation, differentiation, migration, and survival through activation of multiple intracellular signaling pathways [7,8]. Among the most clinically relevant RET alterations, the extracellular C634R mutation is commonly associated with MEN2A syndrome, whereas the kinase-domain M918T mutation represents the molecular hallmark of MEN2B and is associated with particularly aggressive and therapeutically challenging disease forms [5,9]. Closely associated with RET dysregulation, aberrant activation of downstream signaling cascades is involved as well [9,10,11]. Notably, PI3K signaling represents the major downstream effector of RET and plays a central cooperative role in RET-driven MTC progression by sustaining cell survival, tumor growth, metabolic adaptation, and resistance mechanisms in aggressive thyroid malignancies [10,12,13,14].

**Figure 1:**
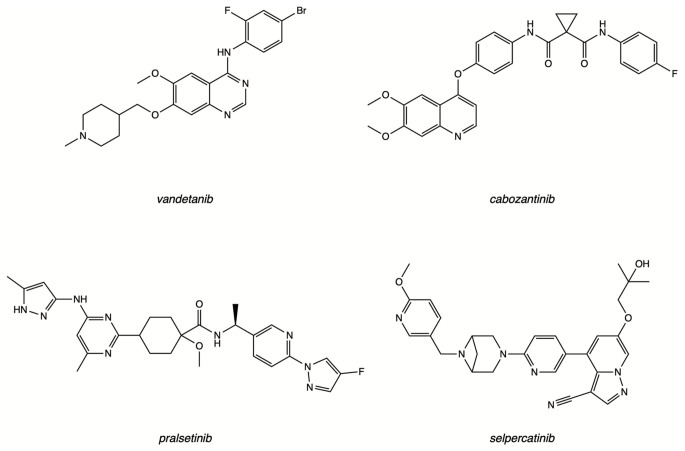
Clinically approved targeted agents used in the treatment of medullary thyroid carcinoma.

In this context, the pharmacological management of advanced and metastatic MTC currently relies on the use of kinase inhibitors with different selectivity profiles. Among the FDA-approved agents, multikinase inhibitors (MKIs) such as vandetanib and cabozantinib, both characterized by quinoline/quinazoline-based scaffolds, together with the more recently developed selective inhibitors selpercatinib and pralsetinib, have significantly improved the treatment of MTC (Figure 1) [4,9,15,16,17]. Despite the significant clinical advances achieved in recent years, currently available treatments still present several limitations, including acquired resistance, bypass signaling activation, tumor heterogeneity, limited long-term efficacy, and clinically relevant adverse effects that may compromise treatment tolerability and therapeutic response [15,16,17]. These limitations continue to stimulate the development of structurally novel chemotypes capable of modulating oncogenic signaling networks involved in aggressive MTC.

**Figure 2:**
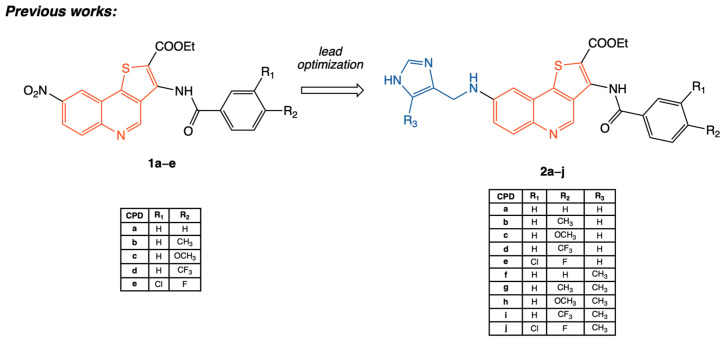
Evolution of the thieno[3,2-c]quinoline scaffold from first-generation derivatives **1a**–**e** active in TT (RET^C634R^) cells to second-generation imidazole-functionalized analogues **2a**–**j** with improved drug-like properties and broad-spectrum antiproliferative activity on NCI-60 panel.

In this context, thieno[3,2-c]quinoline derivatives have attracted growing interest as promising scaffolds for the development of structurally novel targeted anticancer agents [18,19,20,21]. In our previous studies, we identified a first-generation series of thieno[3,2-c]quinoline-2-carboxylates **1** through a ligand-based virtual screening approach (Figure 2). These compounds displayed promising antiproliferative activity (IC_50_ = 3–45 μM) in TT medullary thyroid carcinoma cells harboring the RET^C634R^ mutation, with compound **1b** emerging as the most active derivative of the series (IC_50_ ≈ 3 μM) [22].

To improve the physicochemical and drug-like properties, the first-generation derivatives were subsequently optimized through the introduction of an imidazole moiety according to a molecular hybridization strategy, generating a second-generation series of functionalized thieno[3,2-c]quinolines **2** (Figure 2). In this hybrid architecture, the thieno[3,2-c]quinoline core was combined with the imidazole ring, a privileged scaffold frequently associated with enhanced polarity, hydrogen-bonding capability, and multitarget interaction potential in anticancer drug discovery [23]. The rationally designed library was synthesized and biologically evaluated in the NCI-60 human tumor cell line panel, where the compounds displayed broad-spectrum antiproliferative activity. In addition, complementary in silico target prediction studies and structural similarity analyses suggested phosphoinositide 3-kinase alpha (PI3Kα) as a plausible signaling-associated target connected with the optimized scaffold, revealing significant structural similarities with known PI3Kα inhibitors [23,24,25].

In light of the antiproliferative potential previously observed for the first-generation thieno[3,2-c]quinolines **1** in MTC cellular models, together with those exhibited by the optimized imidazole-functionalized derivatives **2**, in this work we investigated whether lead optimization could improve and expand the biological activity of the scaffold in the context of medullary thyroid carcinoma cells. Considering that derivatives **2** were previously evaluated in the NCI-60 human tumor cell line panel, which does not include thyroid cancer models, the same compounds were herein specifically assessed in two MTC cellular models characterized by distinct RET mutational backgrounds, namely TT(RET^C634R^) cells and the more aggressive MZ-CRC-1(RET^M918T^) model.

In parallel, complementary structure-based computational studies, including induced fit docking, MM-GBSA calculations, molecular dynamics simulations and ADMET analysis were performed in order to investigate the potential interactions of the optimized scaffold with signaling-associated molecular targets involved in MTC pathogenesis and progression, and to explore putative molecular determinants underlying the observed antiproliferative activity.

## 2. Results and Discussion

### 2.1. Synthesis of Thieno[3,2-c]Quinoline Derivatives ***2a***–***j***

The synthesis of the first-generation thieno[3,2-c]quinoline-2-carboxylates **1a**–**e** and the optimized derivatives **2a**–**j** included in the present study was previously reported by our research group [22,23]. Herein, only the key synthetic steps are briefly recalled, while full experimental procedures and characterization data are available in the corresponding references.

Briefly, the nitro-substituted thieno[3,2-c]quinoline derivatives **1a**–**e** were synthesized starting from p-nitroaniline through sequential construction of the quinoline and fused thieno[3,2-c]quinoline scaffolds, followed by final acylation with appropriately substituted benzoyl chlorides to afford the target compounds **1a**–**e** [22].

**Scheme 1:**
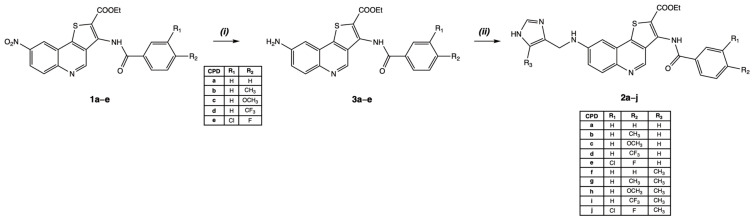
Reagents and conditions: (**i**) 10% Pd/C, H_2_, ethanol, rt 24 h; (**ii**) 4-imidazolecarboxaldehyde or 4-methyl-5-imidazolecarboxaldehyde (1.28 equiv.), NaBH_3_CN (1.28 equiv.), ethanol/AcOH (cat.), rt 2 h.

As outlined in Scheme 1, the derivatives **2a**–**j** were subsequently obtained starting from the corresponding nitro intermediates **1a**–**e**, which were converted into amino derivatives **3a**–**e** by catalytic hydrogenation using H_2_/Pd-C conditions. Introduction of the imidazole moiety was finally achieved by reductive amination with 4-imidazolecarboxaldehyde or 4-methyl-5-imidazolecarboxaldehyde under mild acidic conditions using NaBH_3_CN as reducing agent, affording the target compounds **2a**–**j** in moderate to good yields [23].

### 2.2. In Vitro Antiproliferative Assays on TT(RET^C634R^) and MZ-CRC-1(RET^M918T^) MTC Cells

Compounds **2a**–**j** were evaluated by MTT assay against two human MTC cell lines characterized by distinct RET mutational backgrounds, TT(RET^C634R^) and MZ-CRC-1(RET^M918T^). These cellular models were selected because they represent clinically relevant and highly recurrent forms of MTC associated with MEN2A and MEN2B syndromes, respectively [26]. While the previously reported nitro-substituted derivatives **1a**–**e** displayed moderate antiproliferative activity only in TT cells, the present study was designed to evaluate whether imidazole functionalization could further improve the activity of the scaffold and extend its antiproliferative effects toward the more aggressive MZ-CRC-1 cellular model.

Cells were treated with compounds **2a**–**j** for 3 and 6 days to evaluate both early and prolonged antiproliferative effects. Complete concentration-response curves for all tested compounds are provided in the Supplementary Materials (Figures S1 and S2), whereas the IC_50_ values are reported in Table 1. IC_50_ values of the parent nitro-substituted derivatives **1a**–**e**, previously determined using the same cell lines, treatment times, and MTT-based experimental protocol, were included as reference values to facilitate structure–activity relationship (SAR) comparisons between the two generations of compounds.

As summarized in Table 1, compounds **2a**–**j** displayed in general interesting antiproliferative activity in both MTC cellular models, with several derivatives reaching sub micromolar potency.

In TT(RET^C634R^) cells, compounds **2a**,**b**,**c**,**f**,**g** displayed markedly improved antiproliferative activity compared with the corresponding first-generation nitro-substituted derivatives. Interestingly, the same derivatives had previously emerged among the most active compounds of the series in the NCI-60 human tumor cell line panel.

Among them, compounds **2b** and **2g** emerged as the most active ones, showing IC_50_ values of 0.55 ± 0.3 and 0.64 ± 0.2 μM after 3 days of treatment, respectively. Additional significant activity was observed for the unsubstituted benzoyl derivatives **2a** and **2f** (IC_50_ values of 1.4 ± 0.3 and 1.8 ± 0.4 μM after 3 days), as well as for the p-methoxy-substituted analogue **2c** (IC_50_ value of 1.7 ± 0.1 μM after 3 days), all showing low micromolar or submicromolar IC_50_ values and clear improvement relative to the corresponding parent nitro derivatives reported in Table 1 (IC_50_ = 26.8 ± 2.7 μM for **1a**; IC_50_ = 19.5 ± 9.1 for **1c**, respectively). For some derivatives, reliable IC_50_ determination in TT cells was not possible because of non-ideal concentration–response profiles that prevented accurate curve fitting.

Interestingly, a partially different antiproliferative profile emerged in the more aggressive MZ-CRC-1(RET^M918T^) cellular model, suggesting that subtle structural modifications may differentially influence activity depending on the RET mutational background. In particular, compounds **2d** and **2i** emerged as the most active derivatives (both with a 4-CF_3_ substitution), exhibiting IC_50_ values of 1.3 ± 0.5 and 1.97 ± 0.22 μM after 3 days of treatment, respectively, which further improved after 6 days.

Additional compounds, including **2f**, **2g**, and **2h**, also displayed significant antiproliferative effects, with IC_50_ values in the low micromolar range after both treatment times. In contrast, compounds **2b** and **2c**, among the most active derivatives in TT cells, showed markedly reduced activity in MZ-CRC-1 cells. Moreover, compounds **2e** and **2j** proved essentially inactive in MZ-CRC-1 cells.

### 2.3. Preliminary Structure–Activity Relationship Analysis

**Figure 3:**
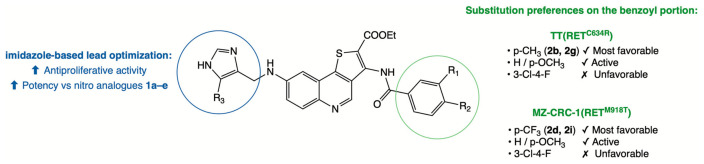
Summary of the main structure–activity relationship (SAR) trends identified for the imidazole-functionalized thieno[3,2-c]quinoline derivatives.

To facilitate the interpretation of the biological results, a preliminary structure–activity relationship (SAR) analysis was performed on the investigated series and is graphically summarized in Figure 3.

Overall, the biological profile of the series appeared strongly influenced by both the substitution pattern on the benzoyl moiety and the cellular context associated with the different RET mutational backgrounds. In particular, the introduction of an imidazole moiety, either unsubstituted (**2a**–**e**) or bearing a 5-methyl substituent (**2f**–**j**), resulted in a substantial improvement and/or extension of the antiproliferative activity compared with the corresponding first-generation nitro derivatives **1a**–**e**, confirming the effectiveness of the adopted lead optimization strategy.

In TT(RET^C634R^) cells, compounds bearing a p-methylbenzoyl substituent (**2b** and **2g**) emerged as the most active derivatives of the series, confirming the favorable effect of this substitution pattern already observed for compound **1b**, the most active member of the first-generation analogues. The unsubstituted benzoyl derivatives (**2a** and **2f**) and the corresponding p-methoxy analogues (**2c** and **2h**) also displayed relevant antiproliferative activity, although generally lower than that observed for the p-methyl-substituted compounds.

A different trend was observed in MZ-CRC-1(RET^M918T^) cells. While imidazole functionalization again produced a clear gain of activity relative to the first-generation nitro derivatives, the highest potency was achieved for compounds bearing a p-trifluoromethylbenzoyl group (**2d** and **2i**), suggesting that this substitution pattern is particularly favorable in the MEN2B-associated cellular model. Conversely, derivatives containing the combined 3-chloro-4-fluoro substitution pattern (**2e** and **2j**) exhibited lower or negligible activity, confirming the unfavourable effect of this structural arrangement previously observed within the parent series. A similarly unfavorable biological trend associated with this substitution pattern had already emerged in the previous NCI-60 screening studies performed on the same scaffold [23].

These findings indicate that both imidazole-based optimization and the nature of the benzoyl substituent play a relevant role in modulating the antiproliferative activity of the thieno[3,2-c]quinoline scaffold, while also highlighting distinct substitution preferences associated with different RET mutational backgrounds.

### 2.4. In Silico Structure-Based Studies

Based on the antiproliferative results and the SAR trends identified across the two MTC cellular models, compounds **2b** and **2g**, which emerged as the most active derivatives in TT(RET^C634R^) cells, together with compounds **2d** and **2i**, identified as the most potent analogues in MZ-CRC-1(RET^M918T^) cells, were selected for subsequent structure-based computational studies. These compounds represent the most promising members of the investigated series and were therefore considered suitable candidates for exploring potential molecular determinants underlying the observed biological activity. The computational analyses were designed to investigate their possible interaction with proliferation-related molecular targets and to provide an initial mechanistic framework capable of guiding future target-validation studies and more detailed investigations.

In light of the central role played by RET and its associated pathways, particularly PI3K signaling, in the proliferation and progression of MTC, explorative structure-based computational studies were performed on selected derivatives of the series. The relevance of these pathways is further supported by previous studies demonstrating that pharmacological inhibition of RET and/or PI3K-related signaling exerts significant antiproliferative effects in both TT and MZ-CRC-1 cellular models [27,28,29]. Moreover, the investigated thieno[3,2-c]quinoline derivatives share relevant structural features and scaffold similarities with several kinase-targeted anticancer agents, including inhibitors associated with RET- and PI3Kα-related pathways [22,23].

Accordingly, the selected best-performing compounds in vitro were initially evaluated against both RET kinase and PI3K kinase targets through induced fit docking (IFD) studies. Subsequently, the most representative derivatives identified from the docking analyses were further investigated by MM-GBSA calculations and molecular dynamics simulations in order to evaluate the stability and persistence of the predicted ligand–target interactions.

#### 2.4.1. Induced Fit Docking Analysis in RET^WT^ and RET^M918T^

Considering the distinct RET mutational backgrounds characterizing TT(RET^C634R^) and MZ-CRC-1(RET^M918T^) cells, together with the different antiproliferative profiles observed for the investigated derivatives, separate induced fit docking (IFD) analyses were performed on two different RET kinase isoforms. In particular, compounds **2b** and **2g**, identified as the most active derivatives in TT cells, and compounds **2d** and **2i**, which emerged as the most potent compounds in MZ-CRC-1 cells, were independently investigated in RET^WT^ and RET^M918T^ kinase domains, respectively.

To reproduce the kinase-domain context associated with TT(RET^C634R^) cells, docking studies were performed on the wild-type RET kinase domain using the crystal structure with PDB code 6NEC [30], co-crystallized with the multikinase inhibitor nintedanib. This approach is justified by the fact that the C634R mutation is located in the extracellular cysteine-rich domain and induces constitutive receptor dimerization without directly altering the ATP-binding site of the intracellular kinase domain, which therefore remains structurally comparable to the wild-type enzyme.

The resulting IFD Score, Prime Energy, and docking score values, used as qualitative parameters to estimate the predicted affinity and interaction profile of the investigated derivatives within the RET ATP-binding pocket, are summarized in Table 2 for compounds **2b** and **2g**, together with those of representative clinically relevant RET inhibitors and reference ligands included for comparison. Compounds **2b** and **2g** displayed highly favorable docking parameters, with IFD Score values of −647.44 and −648.49, respectively. These values were found to be comparable with those obtained for clinically approved RET inhibitors and multikinase inhibitors included in the analysis, such as pralsetinib, selpercatinib, vandetanib, cabozantinib and nintedanib (co-crystallized ligand), as well as the natural substrate ATP. Likewise, both compounds exhibited highly favorable docking scores, suggesting a favorable geometry and accommodation within the RET ATP-binding cleft, with a similar trend observed for Prime Energy values.

Interestingly, the imidazole-thienoquinoline derivatives displayed more favorable predicted interaction parameters relative to the most active first-generation compound **1b**.

Conversely, to investigate the potential interaction of the compounds with a clinically relevant kinase-domain M918T mutant, the second series of IFD calculations performed employed the RET^M918T^ crystal structure (PDB code 4CKI), co-crystallized with adenosine [31]. The corresponding docking results obtained for compounds **2d** and **2i** are summarized in Table 3, which exhibited a similar interesting trend (Table 3). In particular, compound **2i** exhibited an IFD Score value of −667.97, whereas compound **2d** showed a value of −665.49, both comparable with those obtained for clinically relevant RET inhibitors included as reference compounds. Likewise, favorable docking score and Prime Energy values were observed for both derivatives, supporting a favorable predicted interaction profile within the mutated ATP-binding cleft.

Based on the favorable docking profiles obtained in the respective RET kinase domains, compounds **2b** and **2g** in RET^WT^, together with compounds **2d** and **2i** in RET^M918T^, were considered sufficiently interesting for subsequent MM-GBSA calculations aimed at further investigating the stability of the predicted ligand–target interactions.

#### 2.4.2. Induced Fit Docking Analysis in PI3Kα Catalytic Subunit

Additional IFD studies were performed on the catalytic subunit of PI3Kα using the crystallographic structure with PDB code 8EXL, co-crystallized with the PI3Kα inhibitor taselisib [32].

Unlike the RET docking analyses, in which different derivatives were separately evaluated according to the distinct RET mutational backgrounds of the investigated cellular models, all four selected compounds (**2b**,**d**,**g**,**i**) were collectively investigated against PI3Kα, given the recognized involvement of PI3K-associated signaling in both TT and MZ-CRC-1 cells. Compound **1b**, representative of the first-generation series, was also included for comparison.

The resulting IFD Score, Prime Energy, and docking score values, used as qualitative parameters to estimate the predicted affinity and interaction profile of the investigated derivatives within the PI3Kα catalytic pocket, are summarized in Table 4 together with those of representative clinically relevant PI3Kα inhibitors and reference ligands included for comparison.

As summarized in Table 4, among the investigated compounds, derivatives **2b** and **2g** exhibited the most favorable predicted interaction profiles within the selected series, showing IFD Score values of −2039.37 and −2038.77, respectively, comparable to or slightly more favorable than those obtained for the clinically relevant PI3Kα inhibitors omipalisib and dactolisib included as reference compounds. Likewise, highly favorable docking score values were observed, suggesting a favorable geometric accommodation within the ATP-binding region of PI3Kα. Interestingly, the same derivatives generally displayed predicted interaction parameters improved relative to compound **1b**. The obtained computational findings appear consistent with previous in silico target prediction analyses performed on the same scaffold [23,24,25], and with the pronounced antiproliferative activity experimentally observed for the investigated compounds in MTC cellular models.

Conversely, compounds **2d** and **2i** showed interaction profiles closer to compound **1b** and occupied lower positions within the overall docking ranking.

Based on the obtained docking results, compounds **2b** and **2g** were selected for subsequent MM-GBSA calculations on PI3Kα.

#### 2.4.3. Prime MM-GBSA Energy Decomposition Analysis

To gain additional insight into the energetic contributions underlying the predicted ligand–target interaction profiles, Prime MM-GBSA energy decomposition analyses were performed on the best-ranked IFD poses obtained for RET^WT^, RET^M918T^, and PI3Kα complexes. The resulting energetic parameters, including Coulomb, hydrogen-bonding, lipophilic, solvation, and van der Waals contributions, are summarized in Supplementary Materials (Tables S1–S3).

For the RET^WT^ complexes (Table S1), compounds **2b** and **2g** displayed balanced energetic profiles characterized by favorable lipophilic, hydrogen-bonding, solvation and van der Waals contributions, overall consistent with the stable accommodation predicted within the ATP-binding cleft during docking analyses. Interestingly, both optimized derivatives showed interaction profiles improved relative to **1b**, further supporting the beneficial effect of lead optimization on the interaction properties of the scaffold.

A similar energetic trend was observed for compounds **2d** and **2i** in the RET^M918T^ kinase domain (Table S2). In particular, favorable lipophilic, van der Waals and hydrogen-bonding contributions were maintained also within the mutated kinase environment.

Likewise, compounds **2b** and **2g** exhibited favorable energetic profiles also in the PI3Kα catalytic domain (Table S3), with balanced electrostatic, lipophilic, and van der Waals contributions comparable to those observed for the reference PI3K inhibitors included in the analysis. Overall, the MM-GBSA decomposition analyses further supported the selection of compounds **2b** and **2g** for subsequent molecular dynamics simulations on PI3Kα, as well as compounds **2b**, **2d**, **2g**, and **2i** for further dynamic evaluation in the corresponding RET kinase domains.

#### 2.4.4. Molecular Dynamics Simulations and Ligand-Protein Stability Analysis

To further investigate the dynamic stability of the predicted ligand–protein complexes, 200 ns molecular dynamics (MD) simulations were performed on the best-ranked induced fit docking (IFD) poses obtained for the most promising imidazole-functionalized thieno[3,2-c]quinoline derivatives identified through docking analyses and further supported by Prime MM-GBSA calculations. In particular, the selected complexes (**2b**,**g** in complex with RET^WT^, **2d**,**i** with RET^M918T^, **2b**,**g** with PI3Kα and the corresponding reference inhibitors) were subjected to MD simulations in order to evaluate the stability and persistence of the predicted binding modes over time under dynamic conditions.

Initial visual inspection of the generated trajectories indicated that all investigated complexes remained stable throughout the entire simulation time scale. No major structural distortions, large-scale conformational rearrangements of the protein structures, or ligand dissociation events from the binding clefts were observed during the simulations.

To quantitatively evaluate the structural stability of the simulated systems, protein and ligand root mean square deviation (RMSD) profiles were analyzed for all three investigated protein targets in complex with the selected compounds. In parallel, root mean square fluctuation (RMSF) analyses were performed to evaluate local residue flexibility during the simulation trajectories, whereas representative ligand–protein interaction maps were generated in order to visualize the main interactions responsible for ligand accommodation within the catalytic regions.

**Figure 4:**
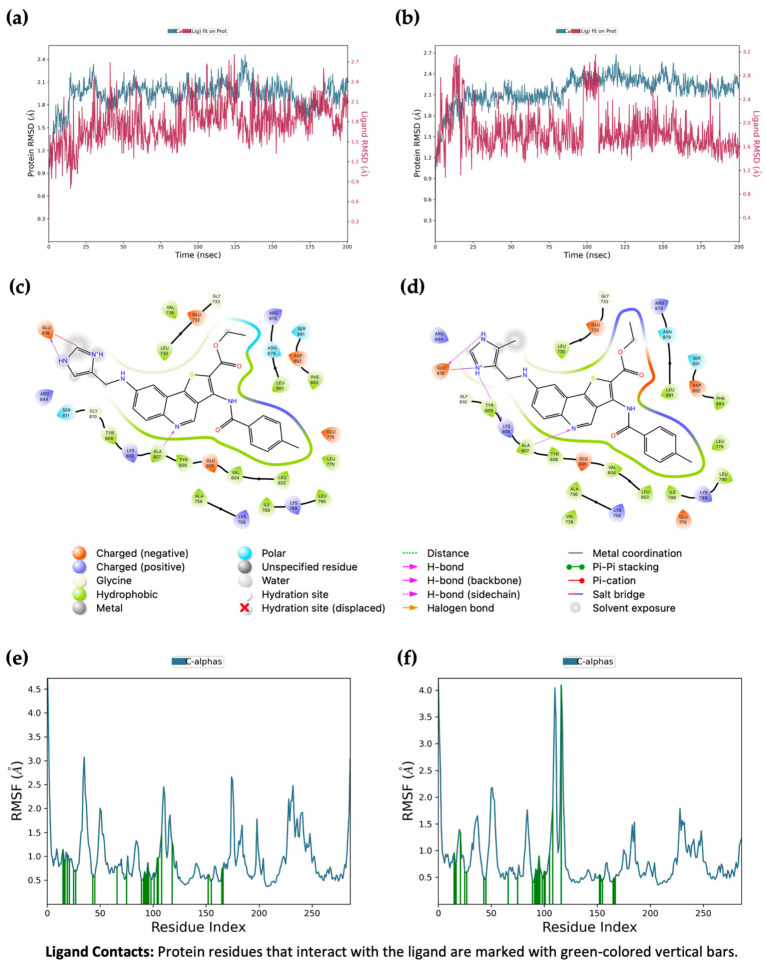
Molecular dynamics simulation analysis of compounds **2b** and **2g** in the RET^WT^ kinase domain (PDB ID: 6NEC). Panels (**a**,**b**) report protein Cα RMSD and ligand RMSD profiles obtained during 200 ns MD simulations for compounds **2b** and **2g**, respectively. Panels (**c**,**d**) show the corresponding representative ligand–protein interaction maps highlighting the main hydrogen-bonding, hydrophobic, polar, and water-mediated contacts established within the ATP-binding cleft. Panels (**e**,**f**) report the RMSF profiles calculated for protein Cα atoms during the simulation trajectories, highlighting local residue flexibility and ligand-contact regions.

Initially, compounds **2b** and **2g**, were investigated in complex with the RET^WT^ kinase domain (PDB ID: 6NEC). Both complexes rapidly reached equilibration and maintained overall stable RMSD profiles throughout the simulations (Figure 4a,b), with fluctuations below the value of 3 Å both for protein and ligands, generally comparable to or lower than those observed for the apo RET^WT^ form and for representative clinically relevant RET inhibitors included as reference systems in the Supplementary Materials (Figure S3).

Representative interaction maps further highlighted the persistence of several hydrophobic, polar, and hydrogen-bonding interactions within the RET catalytic cleft, involving residues located around the ATP-binding region, as Ala807, Lys808 and Glu818 and the residues in the deep hydrophobic cleft (Figure 4c,d). RMSF analyses demonstrated that the main conformational fluctuations were predominantly localized in solvent-exposed and peripheral regions of the kinase domain, whereas residues directly involved in ligand recognition and binding remained comparatively stable throughout the trajectories (Figure 4e,f). Additional RMSF profiles, interaction maps, and interaction fraction analyses performed on representative clinically relevant RET inhibitors, the co-crystallized ligand nintedanib, and reference compound **1b** are reported in the Supplementary Materials (Figures S4–S6), providing a comparative dynamic framework for the investigated derivatives.

Subsequently, compounds **2d** and **2i**, were dynamically investigated in the RET^M918T^ kinase domain (PDB ID: 4CKI). In this case, analysis of the RMSD profiles revealed that the apo RET^M918T^ system displayed overall slightly higher fluctuations compared with the apo RET^WT^ form, suggesting greater intrinsic flexibility of the mutated kinase domain (Supplementary Materials, Figure S7).

**Figure 5:**
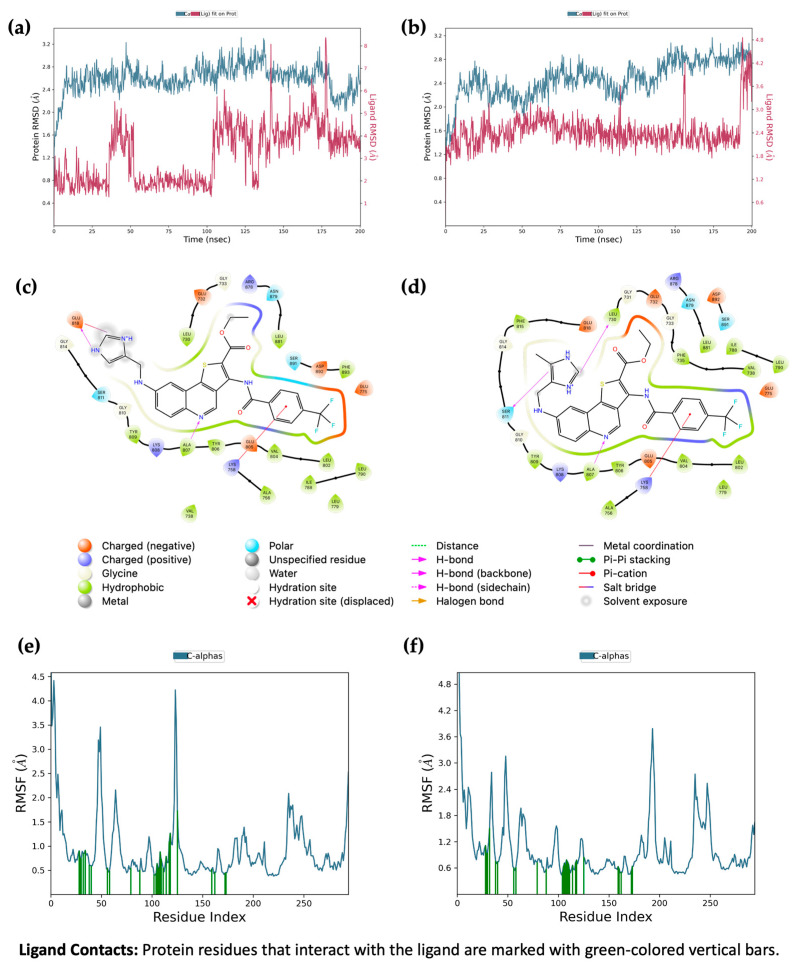
Molecular dynamics simulation analysis of compounds **2d** and **2i** in the RET^M918T^ kinase domain (PDB ID: 4CKI). Panels (**a**,**b**) report protein Cα RMSD and ligand RMSD profiles obtained during 200 ns MD simulations for compounds **2d** and **2i**, respectively. Panels (**c**,**d**) show the corresponding representative ligand–protein interaction maps highlighting the main hydrogen-bonding, hydrophobic, polar, and water-mediated contacts established within the ATP-binding cleft of the mutated kinase domain. Panels (**e**,**f**) report the RMSF profiles calculated for protein Cα atoms during the simulation trajectories, highlighting local residue flexibility and ligand-contact regions.

Despite this increased dynamic behaviour, both ligand-bound systems reached equilibration and maintained stable protein RMSD profiles during the simulations. Among the investigated complexes, compound **2i** exhibited particularly favorable dynamic stability, showing limited ligand RMSD fluctuations and persistent accommodation within the ATP-binding cleft throughout the entire trajectory (Figure 5a,b). Compound **2d** displayed slightly higher conformational mobility characterized by transient fluctuations during the simulation, while still maintaining stable binding within the catalytic region (Figure 5a,b). Consistently, interaction maps highlighted the maintenance of stabilizing hydrophobic and polar contacts within the mutated ATP-binding pocket, as Leu730, Lys758, Ala807, Ser811, Glu818 (Figure 5c,d). Furthermore, RMSF analyses again demonstrated that the highest residue flexibility mainly involved peripheral and solvent-exposed regions rather than residues directly associated with ligand recognition (Figure 5e,f). Additional RMSD profiles, RMSF analyses, interaction maps, and interaction fraction histograms obtained for representative clinically relevant RET inhibitors, the co-crystallized ligand adenosine, and reference compound **1b** are reported in the Supplementary Materials (Figures S7–S10).

**Figure 6:**
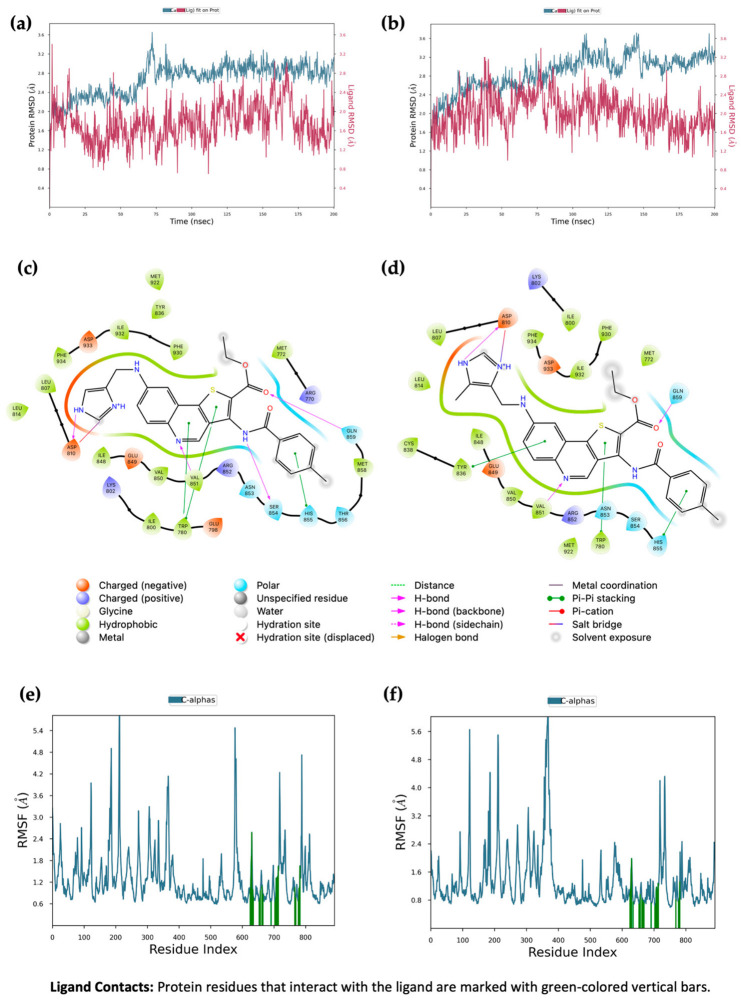
Molecular dynamics simulation analysis of compounds **2b** and **2g** in the PI3Kα catalytic domain (PDB ID: 8EXL). Panels (**a**,**b**) report protein Cα RMSD and ligand RMSD profiles obtained during 200 ns MD simulations for compounds **2b** and **2g**, respectively. Panels (**c**,**d**) show the corresponding representative ligand–protein interaction maps highlighting the principal hydrogen-bonding, hydrophobic, polar, and water-mediated contacts established within the catalytic pocket. Panels (**e**,**f**) report the RMSF profiles calculated for protein Cα atoms during the simulation trajectories, highlighting local residue flexibility and ligand-contact regions.

Considering the favorable docking and MM-GBSA profiles observed for compounds **2b** and **2g** in PI3Kα, additional MD simulations were performed on the corresponding PI3Kα complexes (PDB ID: 8EXL). Both systems displayed stable RMSD trajectories during the entire simulation time scale, with values below 3 Å for both protein and ligands, with no evidence of ligand dissociation from the catalytic pocket (Figure 6a,b). Representative interaction maps highlighted the persistence of several hydrophobic and polar contacts within the PI3Kα catalytic domain (Trp780, Asp810, Tyr836, Val851, Ser854, His855, Gln859) supporting stable accommodation of the investigated derivatives within the ATP-binding region (Figure 6c,d). Likewise, RMSF profiles indicated that the main conformational fluctuations were predominantly associated with flexible peripheral regions of the protein structure, whereas residues involved in ligand recognition remained comparatively stable throughout the simulations (Figure 6e,f).

Additional RMSD profiles, RMSF analyses, interaction maps, and interaction fraction histograms obtained for representative clinically relevant PI3K inhibitors, the co-crystallized ligand taselisib, and reference compound **1b** are reported in the Supplementary Materials (Figures S11–S14), allowing direct comparison between the investigated derivatives and representative reference systems under dynamic conditions.

Overall, the MD simulations consistently supported the structural stability and persistence of the predicted binding modes of the investigated imidazole-functionalized thieno[3,2-c]quinoline derivatives within RET^WT^, RET^M918T^, and PI3Kα catalytic domains.

#### 2.4.5. Predicted ADME and Toxicological Profile of Lead Compounds

To gain preliminary insights into the developability of the most promising derivatives, the physicochemical, pharmacokinetic, and toxicity-related properties of compounds **2b**,**d**,**g**,**i** were evaluated using QikProp, SwissADME, and ProTox-3.0. For comparative purposes, the corresponding data of the previously identified lead compound **1b** were also included (Table 5).

The investigated derivatives displayed physicochemical profiles for the majority compatible with drug-like molecules. Compared with compound **1b**, the new series of compounds resulted in improved predicted oral absorption and reduced structural liabilities, as evidenced by the complete absence of Brenk alerts for compounds **2b**,**d**,**g**,**i**. All selected derivatives showed high predicted Caco-2 permeability and favorable human oral absorption values, while none were predicted to behave as P-glycoprotein substrates, suggesting a potentially advantageous absorption profile.

**Figure 7:**
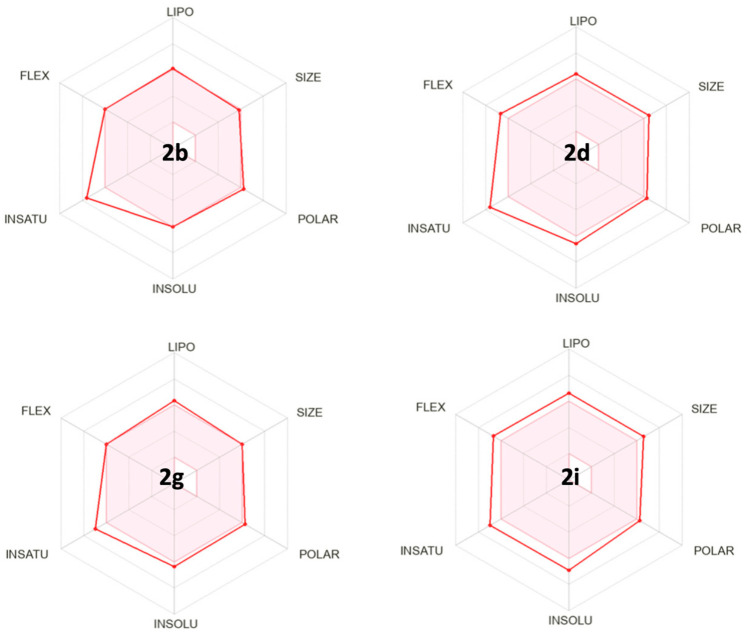
SwissADME bioavailability radar plots of the selected lead compounds **2b**,**d**,**g**,**i**. The radar charts provide a graphical assessment of the main physicochemical properties associated with oral bioavailability (optimal ranges within the pink area), including lipophilicity (LIPO), molecular size (SIZE), polarity (POLAR), solubility (INSOLU), saturation (INSATU), and flexibility (FLEX).

The compounds also exhibited balanced molecular weight, lipophilicity, polarity, and flexibility parameters, with only limited deviations from the classical Lipinski criteria, which is commonly observed for heteroaromatic anticancer agents. Furthermore, no PAINS alerts were detected for any of the investigated compounds, supporting the reliability of the observed biological activities. The SwissADME bioavailability radar plots (Figure 7) further confirmed that the selected derivatives occupy a physicochemical space generally consistent with orally bioavailable drug candidates. As shown in Figure 7, all compounds fall largely within, or very close to, the optimal pink area, indicating a balanced profile in terms of the principal physicochemical properties associated with oral bioavailability. Among the evaluated parameters, saturation and solubility showed the greatest, albeit still moderate, deviations from the recommended range. These aspects will be taken into consideration during future lead optimization efforts aimed at further improving the developability of this scaffold while preserving its promising antiproliferative activity.

Finally, preliminary toxicity assessment in silico indicated a reduction in the number of predicted toxicity-related alerts compared with reference compound **1b**, particularly for compounds **2b** and **2g**, as well as predicted acceptable LD_50_ values (840–1100 mg/kg), corresponding to a moderate toxicity profile and relatively low acute toxicity. No interaction with known off-targets involved in physiological processes and frequently associated with adverse effects was detected. In addition, ProTox-3.0 analysis revealed no relevant predicted interactions with a panel of safety-related off-targets commonly associated with adverse effects (Figure S15).

Taken together, these findings suggest that the lead compounds identified through biological screening combine promising antiproliferative activity with an overall favorable predicted ADME and toxicity profile, thus supporting their further investigation as potential anticancer agents. The complete set of predicted ADME, drug-likeness, and toxicity-related parameters is provided in the Supplementary Materials (Tables S4–S6).

## 3. Materials and Methods

### 3.1. Chemistry

The imidazole-functionalized thieno[3,2-c]quinoline derivatives **1a**–**e** and **2a**–**j** investigated in the present study were synthesized and fully characterized in our previous works. Their preparation, purification, and structural characterization were performed as previously described [22,23]. All compounds assayed in the biological studies were of adequate purity for these investigations, as established by the analytical data described in the original publication. Additional spectroscopic characterization data (^1^H NMR, ^13^C NMR and HRMS) together with the corresponding spectra of the representative reference compound **1b** and lead derivatives **2b**,**d**,**g**,**i** have been included in the Supplementary Materials (Figures S16–S23).

### 3.2. Biological Assays

#### 3.2.1. Cell Culture

The biological evaluation of **2a**–**j** derivatives was performed under the same experimental conditions previously adopted for the parent nitro-substituted series, allowing a comparison of the antiproliferative profiles of the two generations of compounds [22]. In particular, the human medullary thyroid carcinoma (MTC) cell lines TT and MZ-CRC-1, harboring RET^C634R^ and RET^M918T^ mutations, respectively, were used in the present study. TT cells were kindly provided by Prof. F. Frasca (University of Catania, Italy), whereas MZ-CRC-1 cells were obtained from commercially available sources. TT cells were cultured in Ham’s F12 medium (Euroclone, Milan, Italy) supplemented with 10% fetal bovine serum (FBS), 2 mM GlutaMAX, and 1 mM sodium pyruvate. MZ-CRC-1 cells were maintained in Dulbecco’s Modified Eagle Medium (DMEM, Euroclone, Milan, Italy) supplemented with 10% FBS and 2 mM GlutaMAX. All cell lines were routinely maintained at 37 °C in a humidified incubator containing 5% CO_2_.

#### 3.2.2. Cytotoxicity Assay

The antiproliferative activity of the investigated compounds was evaluated through the colorimetric MTT assay according to a previously described procedure with minor modifications [33]. Briefly, TT and MZ-CRC-1 cells were seeded into 96-well plates at an appropriate density in 90 μL of complete culture medium and allowed to attach for 24 h. Subsequently, cells were treated with serial dilutions of the investigated compounds and incubated for additional time periods corresponding approximately to one and two doubling times of the investigated cell lines, i.e., 3 and 6 days, respectively. Similar incubation intervals are commonly employed in literature studies involving medullary thyroid carcinoma cell models and allow the evaluation of both early and prolonged antiproliferative responses [22,34,35,36,37,38]. At the end of the treatment period, 10 μL of MTT solution (5 mg/mL in PBS; Sigma-Aldrich, Milan, Italy) were added to each well, followed by incubation for 4 h at 37 °C. The resulting insoluble formazan crystals were dissolved in DMSO, and absorbance values were recorded using a Multiskan FC microplate reader (Thermo Fisher Scientific, Milan, Italy) at 550 nm using 620 nm as reference wavelength. Cell viability was expressed as percentage relative to untreated control cells. IC_50_ values were calculated using GraphPad Prism software 9.5.0 (GraphPad Software Inc., San Diego, CA, USA).

#### 3.2.3. Statistical Analysis

Data are expressed as mean ± standard deviation (SD). For MTT assays, cells were seeded in quadruplicate wells for each concentration, and experiments were independently repeated twice. Dose–response curves and IC_50_ values were calculated by nonlinear regression using a four-parameter logistic model (Hill equation) implemented in GraphPad Prism software 9.5.0 (GraphPad Software Inc., San Diego, CA, USA). Statistical comparisons were performed using one-way analysis of variance (ANOVA), and differences were considered statistically significant at *p* < 0.05.

### 3.3. In Silico Studies

#### 3.3.1. Ligand Preparation

All compounds included in the computational study were prepared using the LigPrep module available in the Maestro graphical interface (Schrödinger Release 2017-2, Schrödinger LLC, New York, NY, USA) [39]. This procedure was employed to generate energetically optimized three-dimensional structures suitable for subsequent structure-based calculations. For each molecule, possible ionization and tautomeric states, stereoisomers, and relevant ring conformations were automatically generated while preserving the eventual stereochemical information specified in the input structures. Water molecules, counterions, and other extraneous species were removed during the preparation process. Protonation states were assigned using the Epik algorithm within a physiological pH range of 7.0 ± 0.4. All resulting structures were then subjected to geometry optimization using the OPLS 2005 force field [40], and the corresponding minimized low-energy conformations were used in all subsequent docking, Induced Fit Docking, MM-GBSA, and molecular dynamics simulations.

#### 3.3.2. Protein Preparation

The crystallographic structures of the proteins investigated in this study were retrieved from the Protein Data Bank in .pdb format [41,42]. The following structures were used: RET^WT^ (PDB id: 6NEC) [30], RET^M918T^ (PDB id: 4CKI) [31], PI3Kα (PDB id: 8EXL) [32]. All protein structures were prepared using the Protein Preparation Wizard implemented in the Maestro interface (Schrödinger Release 2017-1, Schrödinger LLC, New York, NY, USA) [40,43,44]. During preprocessing, bond orders were assigned, hydrogen atoms were added, and crystallographic water molecules and other non-essential hetero groups were removed. Protonation states were assigned using the Epik module at pH 7.0 ± 0.4, and disulfide bonds were automatically identified. Missing side chains and loops located in proximity to the ligand-binding site were reconstructed using the Prime module when necessary.

The hydrogen-bonding network was subsequently optimized, and all structures were subjected to restrained energy minimization with the OPLS 2005 force field [40], until convergence was reached with a maximum heavy-atom root-mean-square deviation (RMSD) of 0.3 Å. The resulting optimized protein models were used for all subsequent docking, induced fit docking, MM-GBSA, and molecular dynamics simulations.

#### 3.3.3. Structure-Based Studies: Induced Fit Docking (IFD) Simulations on RET and PI3Kα X-Ray Structures

Induced Fit Docking (IFD) calculations were performed on RET^WT^ (PDB id: 6NEC) [30], RET^M918T^ (PDB id: 4CKI) [31], PI3Kα (PDB id: 8EXL) [32], using the Induced Fit Docking protocol implemented in the Maestro interface (Schrödinger Release 2017-1, Schrödinger LLC, New York, NY, USA) [45], following procedures and settings previously adopted by us [46,47]. In details, this approach combines the sampling capabilities of Glide with sidechain and local backbone refinement by Prime, allowing simultaneous adaptation of both the ligand and the receptor and providing a more realistic description of protein–ligand recognition.

The protein structures previously prepared with the Protein Preparation Wizard were used as receptor models. For each ligand, docking poses were generated and subsequently refined through iterative optimization of residues surrounding the binding site under the default settings of the Schrödinger IFD workflow.

The resulting complexes were ranked according to the IFD Score, which integrates the Glide docking score with the corresponding Prime energy according to the relationship: IFD Score = 1.0 × Glide GScore + 0.05 × Prime Energy. This composite score was used together with the associated Prime Energy and XP Docking Score values to compare the predicted binding affinities of the investigated compounds [48,49].

The reliability of the protocol was assessed by redocking the co-crystallized ligands into their respective binding sites. In all cases, the experimental binding modes were accurately reproduced, with root-mean-square deviation (RMSD) values below 0.52 Å, confirming the robustness of the adopted procedure.

#### 3.3.4. Binding Free Energy MM-GBSA Calculations

The binding affinities of the selected protein–ligand complexes were further assessed by molecular mechanics with generalized Born and surface area (MM-GBSA) calculations using the Prime module implemented in the Maestro interface (Schrödinger Release 2017-1, Schrödinger LLC, New York, NY, USA) [50]. The best-ranked complexes obtained from the induced fit docking (IFD) studies were used as input structures.

Binding free energies were estimated according to the single-trajectory approach implemented in Prime, which combines molecular mechanics energies with implicit solvation effects to provide a relative estimate of protein–ligand interaction strength. Calculations were performed using the VSGB solvation model with water as solvent and the OPLS 2005 force field under the default settings.

In addition to the overall Prime Energy, the energetic decomposition provided by the Prime MM-GBSA protocol was analyzed to gain further insight into the factors governing ligand recognition. The reported terms included Coulombic interactions (Prime Coulomb), hydrogen-bonding contributions (Prime Hbond), lipophilic interactions (Prime Lipo), packing interactions (Prime Packing), intramolecular self-contact corrections (Prime SelfCont), polar solvation effects (Prime Solv GB), and van der Waals interactions (Prime vdW). These descriptors were used for comparative evaluation of the binding profiles of the synthesized compounds relative to the corresponding co-crystallized and clinically approved reference inhibitors.

#### 3.3.5. Molecular Dynamics (MD) Simulations

Molecular dynamics (MD) simulations were performed to investigate the dynamic stability of the selected protein–ligand complexes in an explicit solvent environment using the Desmond MD engine implemented in the Maestro interface (Schrödinger Release 2023-4; Desmond version 13.8.155, MMshare version 6.4.195) [51]. Initial coordinates were taken from the best-ranked induced fit docking poses and processed with the System Builder module.

Each system was embedded in an orthorhombic simulation box filled with SPC water molecules, maintaining a minimum distance of 10 Å between the solute and the box boundaries. Appropriate counterions (Na^+^ or Cl^−^) were added to neutralize the overall system charge. All simulations were carried out using the OPLS4 force field. After energy minimization and the default multistep relaxation protocol provided by Desmond, production simulations were performed under periodic boundary conditions in the isothermal–isobaric (NPT) ensemble at 300 K and 1.01325 bar.

All ligand–protein complexes were simulated for 200 ns. In parallel, 200 ns molecular dynamics simulations were also carried out on the prepared apo forms of RET^WT^, RET^M918T^ and PI3Kα using the same protocol. These simulations served as reference models to characterize the intrinsic dynamics of the unliganded proteins and to assess the stabilizing effects of ligand binding. Approximately 1000 trajectory frames were saved for each simulation and used for post-processing analyses.

Trajectory analysis was carried out using the Simulation Interaction Diagram and Trajectory Analysis tools available in Maestro. System stability was monitored by examining total and potential energy, temperature, pressure, and volume throughout the simulations. Structural stability was primarily assessed through root-mean-square deviation (RMSD) of protein backbone atoms and ligand heavy atoms, while residue flexibility was evaluated by root-mean-square fluctuation (RMSF). Additional descriptors, including protein–ligand contact persistence, radius of gyration, solvent-accessible surface area (SASA), and ligand torsional behaviour, were analyzed when appropriate to further characterize the stability and conformational evolution of the simulated complexes.

To further assess the robustness and reproducibility of the molecular dynamics results, additional independent 200 ns simulations were performed for the lead compounds **2b**,**d**,**g**,**i** in complex with their respective protein targets, using the same simulation protocol but a different random seed for the assignment of the initial atomic velocities. In addition, supplementary 50 ns simulations were carried out for the same protein–ligand complexes to further evaluate the reproducibility of the predicted binding modes and the stability of the main protein–ligand interactions. All additional trajectories were analyzed using the same workflow adopted for the primary simulations. RMDS graphs for replicate simulations are provided in the Supplementary Materials (Figures S24 and S25).

#### 3.3.6. In Silico ADME and Toxicity Prediction

The physicochemical and pharmacokinetic properties of the lead compounds **2b**,**d**,**g**,**i** were predicted using the QikProp module (Release 2017-1, Schrödinger LLC, New York, NY, USA), available within the Schrödinger Maestro Suite (Maestro Version 11.1.012, Release 2017-1). Prior to ADME calculations, all structures were prepared using the LigPrep workflow under the same conditions adopted for the molecular modeling studies. QikProp was subsequently employed using standard settings to estimate descriptors related to absorption, distribution, metabolism, and excretion (ADME).

Additional physicochemical parameters, drug-likeness descriptors, and bioavailability radar plots were obtained using the SwissADME web server by submitting the SMILES representation of each compound. Compliance with Lipinski’s rule of five and other drug-likeness filters was also assessed [52].

Preliminary toxicity predictions were performed using the ProTox-3.0 web server [53,54,55]. The SMILES representation of compounds **2b**,**d**,**g**,**i** was submitted to obtain an initial assessment of their toxicological profile.

## 4. Conclusions

The present study demonstrates that imidazole-based lead optimization successfully enhanced the biological profile of the thieno[3,2-c]quinoline scaffold against clinically relevant MTCa models. Compared with the previously reported nitro-substituted analogues **1**, several second-generation derivatives displayed markedly improved antiproliferative activity, with compounds **2b** and **2g** emerging as the most active derivatives in TT(RET^C634R^) cells, whereas compounds **2d** and **2i** exhibited the most favorable activity profile in the more aggressive MZ-CRC-1(RET^M918T^) model. Preliminary SAR analysis indicated that imidazole functionalization substantially improved biological activity, while the nature of the benzoyl substituent influenced the activity profile according to the RET mutational background, with p-methylbenzoyl substitution being favored in TT cells and p-trifluoromethylbenzoyl substitution proving optimal in MZ-CRC-1 cells.

Complementary computational studies supported RET, both WT and mut, as the most plausible molecular target for all the selected derivatives (**2b**,**d**,**g**,**i**), while compounds **2b** and **2g** also exhibited favorable interaction profiles toward PI3Kα, suggesting that simultaneous modulation of RET- and PI3K-associated signaling may contribute to the biological activity of selected compounds. Nevertheless, dedicated mechanistic investigations will be required to experimentally establish the relative contribution of these pathways. Finally, the lead compounds exhibited an overall favorable predicted developability profile, combining acceptable physicochemical and pharmacokinetic properties, moderate predicted acute toxicity, reduced toxicity-related alerts compared with the reference nitro derivative, and no significant predicted off-target liabilities.

Collectively, these findings identify compounds **2b**,**d**,**g**,**i** as promising lead structures for the future development of targeted therapeutic agents against aggressive medullary thyroid carcinoma and provide a solid framework for further biological validation and lead optimization.

## Figures and Tables

**Table 1:** Antiproliferative activity (IC_50_) of the imidazole-functionalized thieno[3,2-c]quinolines **2a**–**j** in TT and MZ-CRC-1 cells after 3 and 6 days of treatment, as determined by MTT assay. Previously reported IC_50_ values of the parent derivatives **1a**–**e** were included as reference values.

Compound	R_1_	R_2_	R_3_	TT (RET^C634R^) *		MZ-CRC-1 (RET^M918T^) *	
				3 Days	6 Days	3 Days	6 Days
**2a**	H	H	H	1.4 ± 0.3	0.57 ± 0.1	9.33 ± 1.5	5.61 ± 0.6
**2b**	H	CH_3_	H	0.55 ± 0.3	0.38 ± 0.1	>100	47 ± 6.3
**2c**	H	OCH_3_	H	1.7 ± 0.1	1.5 ± 0.1	68.3 ± 5	49.9 ± 0.6
**2d**	H	CF_3_	H	N.D.	N.D.	1.3 ± 0.5	0.8 ± 0.05
**2e**	Cl	F	H	N.D.	N.D.	>100	>100
**2f**	H	H	CH_3_	1.8 ± 0.4	0.56 ± 0.2	4.1 ± 0.8	3.82 ± 0.5
**2g**	H	CH_3_	CH_3_	0.64 ± 0.2	1.1 ± 0.1	4.4 ± 0.6	6.1 ± 1
**2h**	H	OCH_3_	CH_3_	6.8 ± 1.1	4.1 ± 1	4.7 ± 0.7	5.7 ± 0.8
**2i**	H	CF_3_	CH_3_	N.D.	N.D.	1.97 ± 0.22	1.44 ± 0.35
**2j**	Cl	F	CH_3_	7.7 ± 1.8	4.5 ± 0.9	>100	>100
**1a ^#^**	H	H	-	26.8 ± 2.7	24.3 ± 2.7	-	-
**1b ^#^**	H	CH_3_	-	3.6 ± 0.22	3.01 ± 0.035	-	-
**1c ^#^**	H	OCH_3_	-	19.5 ± 9.1	11.7 ± 4.2	-	-
**1d ^#^**	H	CF_3_	-	73.2 ± 0.002	44.9 ± 5.2	-	-
**1e ^#^**	Cl	F	-	>100	>100	-	-

* IC_50_ values are expressed as mean ± SD from two independent experiments performed in quadruplicate. N.D.: not determined. ^#^ IC_50_ for compounds **1a**–**e** were previously reported in ref [22] and were obtained using the same cell lines, treatment schedule, and MTT-based experimental protocol adopted in the present study.

**Table 2:** Induced Fit Docking (IFD) results obtained for the selected derivatives **2b**,**g** in the wild-type RET kinase domain (PDB ID: 6NEC). Compound **1b**, representative of the first-generation series, together with selected clinically relevant RET inhibitors and reference ligands, were included for comparison. Reported parameters include IFD Score, Prime Energy, and Docking Score.

RET^WT^ (6NEC)			
Compound	IFD Score	Prime Energy	Docking Score
pralsetinib	−653.59	−12,868.5	−10.17
selpercatinib	−650.37	−12,796.2	−10.48
**2g**	−648.49	−12,715.6	−12.68
vandetanib	−647.56	−12,784.8	−8.32
**2b**	−647.44	−12,717.4	−11.48
ATP	−644.74	−12,722.9	−8.52
cabozantinib	−643.89	−12,711.7	−8.22
nintedanib (co-cryst)	−643.80	−12,656.9	−10.56
**1b**	−642.15	−12,678.1	−8.24

**Table 3:** Induced Fit Docking (IFD) results obtained for the selected derivatives **2d**,**i** in the RET^M918T^ kinase domain (PDB ID: 4CKI). Compound **1b**, representative of the first-generation series, together with selected clinically relevant RET inhibitors and reference ligands, were included for comparison. Reported parameters include IFD Score, Prime Energy, and Docking Score.

RET^M918T^ (PDB ID: 4CKI)			
Compound	IFD Score	Prime Energy	Docking Score
pralsetinib	−670.98	−13,223.7	−9.80
**2i**	−667.97	−13,099.5	−12.97
vandetanib	−667.26	−13,160.8	−9.22
selpercatinib	−667.24	−13,140.0	−10.17
**2d**	−665.49	−13,078.2	−11.49
ATP	−664.72	−13,099.3	−9.68
**1b**	−663.09	−13,057.8	−10.20
cabozantinib	−662.26	−13,077.2	−8.32
adenosine (lig-cocryst)	−659.98	−13,011.7	−9.39

**Table 4:** Induced Fit Docking (IFD) results obtained for the selected derivatives **2b**,**d**,**g**,**i** in the PI3Kα catalytic subunit (PDB ID: 8EXL). Compound **1b**, representative of the first-generation series, together with selected clinically relevant PI3Kα inhibitors, were included for comparison. Reported parameters include IFD Score, Prime Energy, and Docking Score.

PI3Kα			
Compound	IFD Score	Prime Energy	Docking Score
**2b**	−2039.37	−40,559.7	−11.30
**2g**	−2038.77	−40,551.5	−11.16
omipalisib	−2038.10	−40,558.1	−10.10
**2d**	−2037.18	−40,516.9	−11.25
**1b**	−2037.14	−40,512.1	−11.53
**2i**	−2036.16	−40,526.8	−9.79
dactolisib	−2035.79	−40,494.5	−11.06
taselisib (lig. co-cryst)	−2034.85	−40,506.2	−9.53

**Table 5:** Predicted physicochemical, ADME and toxicity-related properties for compounds **2b**,**d**,**g**,**i** and **1b**.

Compound	1b	2b	2d	2g	2i
MW *	435.45	486.56	539.53	499.59	553.56
**Csp^3^**	0.14	0.15	0.15	0.19	0.19
**RB**	7	9	10	9	10
**HBA**	6	4	7	4	7
**HBD**	1	4	4	4	4
**TPSA**	142	138	138	138	138
**LogP**	2.50	2.36	2.66	2.56	2.86
**rtvFG**	0	0	0	0	0
**PP-Caco2**	90.11	162.94	178.56	212.68	200.17
**%HOA**	84.44	92.20	70.62	96.90	75.00
**P-gp substrate**	No	No	No	No	No
**LV**	0	0	1	1	1
**PAINS**	0	0	0	0	0
**Brenk alerts**	2	0	0	0	0
**Tox alerts**	9	4	6	4	6

* Abbreviations: MW, molecular weight (Da); Csp^3^, fraction of sp^3^-hybridized carbon atoms; RB, number of rotatable bonds; HBA, hydrogen-bond acceptors; HBD, hydrogen-bond donors; TPSA, topological polar surface area (Å^2^); LogP, predicted octanol/water partition coefficient; rtvFG, reactive functional groups; PP-Caco2, predicted Caco-2 cell permeability (nm/s); HOA, predicted human oral absorption (%); P-gp, P-glycoprotein; LV, Lipinski rule-of-five violations; PAINS, pan-assay interference compounds alerts; Brenk alerts, structural alerts associated with potentially problematic chemical features; Tox alerts, predicted toxicity-related structural alerts.

## Data Availability

The original contributions presented in this study are included in the article/Supplementary Materials. Further inquiries can be directed to the corresponding author.

## Supplementary Materials

The following supporting information can be downloaded at: https://www.mdpi.com/article/10.3390/ph19071037/s1. Supporting Figures: Figure S1. Dose–response curves of compounds **2a**–**j** in TT (RET^C634R^) cells. Figure S2. Dose–response curves of compounds **2a**–**j** in MZ-CRC-1 (RET^M918T^) cells. Figure S3. RET^WT^ (6NEC) RMSD Profiles for reference ligands and apo protein. Figure S4. RET^WT^ (6NEC) RMSF Profiles for reference ligands and apo protein. Figure S5. RET^WT^ (6NEC) 2D interaction maps for reference ligands. Figure S6. RET^WT^ (6NEC) interaction fraction histogram for reference ligands. Figure S7. RET^M918T^ (4CKI) RMSD Profiles for reference ligands and apo protein. Figure S8. RET^M918T^ (4CKI) RMSF Profiles for reference ligands and apo protein. Figure S9. RET^M918T^ (4CKI) 2D interaction maps for reference ligands. Figure S10. RET^M918T^ (4CKI) interaction fraction histogram for reference ligands. Figure S11. PI3Kα (8EXL) RMSD Profiles for reference ligands and apo protein. Figure S12. PI3Kα (8EXL) RMSF Profiles for reference ligands and apo protein. Figure S13. PI3Kα (8EXL) 2D interaction maps for reference ligands. Figure S14. PI3Kα (8EXL) interaction fraction histogram for reference ligands. Figure S15. Predicted toxicity and off-target interaction profiles of **2b**,**d**,**g**,**i**. Figures S16–S23. ^1^H and ^13^CNMR spectra for representative compounds **2b**,**d**,**g**,**i**. Figures S24. RMSD profiles from independent 200 ns molecular dynamics simulations of the lead protein–ligand complexes. Figure S25. RMSD profiles from independent 50 ns molecular dynamics simulations of the lead protein–ligand complexes. Supporting Tables: Table S1. Prime MM-GBSA energy decomposition analysis in RET^WT^ (6NEC). Table S2. Prime MM-GBSA energy decomposition analysis in RET^M918T^ (4CKI). Table S3. Prime MM-GBSA energy decomposition analysis in PI3Kα (8EXL). Table S4–S6. Complete In Silico ADME, Drug-Likeness, and Toxicity Assessment of the Selected Lead Compounds.

## Abbreviations

The following abbreviations are used in this manuscript:

ADME-Tox	absorption, distribution, metabolism, excretion and toxicity
ATP	adenosine triphosphate
CLD	cadherin-like domain
CRD	cysteine-rich domain
FDA	Food and Drug Administration
FMTC	familial medullary thyroid cancer
IC50	half maximal inhibitory concentration
IFD	induced fit docking
MD	molecular dynamics
MEN2	multiple endocrine neoplasia type 2
MKI	multikinase inhibitor
MM-GBSA	molecular mechanics generalized Born surface area
MTC	medullary thyroid carcinoma
MTT	3-(4,5-dimethylthiazol-2-yl)-2,5-diphenyltetrazolium bromide
PDB	Protein Data Bank
PI3K	phosphoinositide 3-kinase
RET	rearranged during transfection
RETC634R	RET C634R mutant
RET^M918T^	RET M918T mutant
RET^WT^	wild-type RET
RMSD	root mean square deviation
RMSF	root mean square fluctuation
SAR	structure–activity relationship
TKD	tyrosine kinase domain
TM	transmembrane domain
WT	wild type
