# Supplementary material for: Imidazole-Functionalized Thieno[3,2-c]Quinoline Hybrids in Aggressive Medullary Thyroid Cancer Cell Models: Biological Evaluation and in Silico Insights
Source: Pharmaceuticals (Basel). 2026 Jul 3;19(7):1037. doi: 10.3390/ph19071037 (PMC13415440; doi:10.3390/ph19071037)
Supplement: Supplementary file 1 [file pharmaceuticals-19-01037-s001.zip › Supporting Material for Pharmaceuticals revised.pdf]

Supporting Material

Article

Imidazole-Functionalized Thieno[3,2-c]Quinoline Hybrids in Aggressive Medullary Thyroid Cancer Cell Models: Biological Evaluation and in Silico Insights

Gabriele La Monica 1,*, Alessia Bono 1,2, Federica Alamia 1, Dennis Tocco 1, Giuseppe Pizzolanti 3,4, Antonino Lauria 1 and Annamaria Martorana 1

1 Dipartimento di Scienze e Tecnologie Biologiche Chimiche e Farmaceutiche "STEBICEF", University of Palermo, 90123 Palermo, Italy; alessia.bono01@unipa.it (A.B.); federica.alamia01@unipa.it (F.A.); dennis.tocco@unipa.it (D.T.); antonino.lauria@unipa.it (A.L.); annamaria.martorana@unipa.it (A.M.)

2 Fondazione Umberto Veronesi (FUV), Via Solferino 19, 20121 Milano, Italy3 Department of Health Promotion, Mother and Child Care, Internal Medicine and Medical Specialties (PROMISE), University of Palermo, 90127 Palermo, Italy; giuseppe.pizzolanti@unipa.it

4 Advanced Technologies Network Center (ATEN Center), University of Palermo, 90128 Palermo, Italy

* Correspondence: gabriele.lamonica01@unipa.it

Table of contents of Supporting Material

Experimental procedures for compound 2a–j

synthesis Supporting Figures:

- **Figure S1.** Dose–response curves of compounds **2a–j** in TT (RET^{C634R}) cells.
- **Figure S2.** Dose–response curves of compounds **2a–j** in MZ-CRC-1 (RET^{M918T}) cells.
- **Figure S3.** RET^{WT} (6NEC) RMSD profiles for reference ligands and apo protein.
- **Figure S4.** RET^{WT} (6NEC) RMSF profiles for reference ligands and apo protein.
- **Figure S5.** RET^{WT} (6NEC) 2D interaction maps for reference ligands.
- **Figure S6.** RET^{WT} (6NEC) interaction fraction histogram for reference ligands.
- **Figure S7.** RET^{M918T} (4CKI) RMSD profiles for reference ligands and apo protein.
- **Figure S8.** RET^{M918T} (4CKI) RMSF profiles for reference ligands and apo protein.
- **Figure S9.** RET^{M918T} (4CKI) 2D interaction maps for reference ligands.
- **Figure S10.** RET^{M918T} (4CKI) interaction fraction histogram for reference ligands.
- **Figure S11.** PI3Kα (8EXL) RMSD profiles for reference ligands and apo protein.
- **Figure S12.** PI3Kα (8EXL) RMSF profiles for reference ligands and apo protein.
- **Figure S13.** PI3Kα (8EXL) 2D interaction maps for reference ligands.
- **Figure S14.** PI3Kα (8EXL) interaction fraction histogram for reference ligands.
- **Figure S15.** Predicted toxicity and off-target interaction profiles of **2b,d,g,i**.
- **Figures S16–23.** ^{1}H and ^{13}CNMR spectra for representative compounds **2b,d,g,i**.
- **Figures S24.** RMSD profiles from independent 200 ns molecular dynamics simulations of the lead protein–ligand complexes.

- **Figure S25.** RMSD profiles from independent 50 ns molecular dynamics simulations of the lead protein–ligand complexes.

Supporting Tables:

- **Table S1.** Prime MM-GBSA energy decomposition analysis in RET^{WT} (6NEC).
- **Table S2.** Prime MM-GBSA energy decomposition analysis in RET^{M918T} (4CKI).
- **Table S3.** Prime MM-GBSA energy decomposition analysis in PI3Kα (8EXL).
- **Table S4–6.** Complete In Silico ADME, Drug-Likeness, and Toxicity Assessment of the Selected Lead Compounds (attached as separated .xlsx file).

Supplementary experimental details for compound synthesis

General information

The thieno[3,2-c]quinoline derivatives investigated in the present study were synthesized according to procedures previously reported by our group [1,2]. The spectroscopic characterization data reported below are provided to confirm the identity and purity of the investigated compounds and were found to be fully consistent with those previously described in the original publications. All chemicals and solvents were purchased from commercial suppliers and used without further purification unless otherwise stated. Melting points were determined in open capillaries using a Büchi Tottoli apparatus and are uncorrected. ^{1}H and ^{13}C NMR spectra were recorded on a Bruker AC-E 400 MHz spectrometer using $CDCl_3$ or DMSO-d_6 as solvents. Chemical shifts (δ) are reported in ppm relative to tetramethylsilane (TMS) as internal standard. Signal multiplicities are designated as follows: br s (broad singlet), s (singlet), d (doublet), t (triplet), q (quartet), and m (multiplet). High-resolution mass spectra (HRMS) were acquired on an Agilent 6540 UHD accurate-mass quadrupole time-of-flight (Q-TOF) instrument. The purity of all compounds evaluated in the biological assays was confirmed to be ≥95% by HPLC/MS analysis.

The synthetic procedures describing the conversion of the previously reported nitro derivatives **1a–e** into the corresponding imidazole-functionalized analogues **2a–j** are reported below. The spectroscopic characterization data of compounds **2a–j** are subsequently provided. Representative ^{1}H and ^{13}C NMR spectra of compounds **2b,d,g,i** are also included in the Supporting Figures section.

*General procedure for the synthesis of ethyl 8-amino-3-benzoylaminoothieno[3,2-c]quinoline-2-carboxylate **3a-e***

A suspension of the corresponding 5-nitro derivatives **1a–e** (1.0 equiv.) and 10% Pd/C (0.05 g) in ethanol was subjected to catalytic hydrogenation using a Parr hydrogenation apparatus (500 psi H_2) for 24 h. Upon completion of the reaction, the mixture was heated, and the catalyst was removed by filtration. The filtrate was concentrated under reduced pressure, and the resulting amino derivatives were obtained after recrystallization from ethyl acetate/ethanol. The synthesized amino intermediates were previously reported and fully characterized by our group. Detailed spectroscopic data are available in the original publication [1].

*General procedure for the synthesis of ethyl 8-(((1H-imidazol-4-yl)methyl)amino)-3-benzoylamino-thieno[3,2-c]quinoline-2-carboxylates (**2a–j**)*

The target imidazole-functionalized thieno[3,2-c]quinoline derivatives **2a–j** were synthesized according to the procedure previously reported by our group. Briefly, 8-amino-thieno[3,2-c]quinoline intermediates **3a–e** (1 eq.) were subjected to reductive amination with the appropriate imidazole aldehyde (4-imidazolecarboxaldehyde or 4-methyl-5-imidazolecarboxaldehyde, 1.28 eq.) in ethanol under mildly acidic conditions, using sodium cyanoborohydride as reducing agent (1.28 eq.). After completion of the reaction, the crude products were purified by flash chromatography and recrystallization with Et_2O to afford the desired compounds.

Full experimental procedures and characterization data have been previously reported in the original publication. The spectroscopic and analytical data reported below are fully consistent with those previously published and are provided herein for completeness. [2].

Ethyl 3-benzoylamino-8-(((1H-imidazol-4-yl)methyl)amino)-thieno[3,2-c]quinoline-2-carboxylate (**2a**)

Yield 45%. Mp 145–147 °C. ^{1}H NMR (DMSO-d_6) δ: 1.25 (t, 3H, J = 7.1 Hz, CH_3), 4.26–4.39 (m, 4H, 2×CH_2), 6.77 (t, 1H, J = 5.4 Hz, NH), 7.03 (d, 1H, J = 2.6 Hz, H-9), 7.07 (s, 1H, H-5″), 7.33 (dd, 1H, J = 9.1, 2.5 Hz, H-7), 7.57–7.70 (m, 4H, H-3′, H-4′, H-5′, H-2″), 7.85 (d, 1H, J = 9.0 Hz, H-6), 8.06–8.14 (m, 2H, H-2′, H-6′), 8.85 (s, 1H, H-4), 10.68 (s, 1H, NH), 11.92 (br s, 1H, NH). ^{13}C NMR (DMSO-d_6) δ: 14.5 (CH_3), 39.4 (CH_2), 61.9 (CH_2), 98.5 (CH), 121.4, 121.6 (CH), 124.8, 128.4 (CH), 129.1 (CH), 129.6, 131.0 (CH), 132.7 (CH), 134.0, 135.5 (CH), 137.5, 138.1, 141.7, 141.9 (CH), 148.6, 162.0, 166.1. HRMS-ESI [$(M+H)^+$]: m/z calculated for $C_{25}H_{21}N_5O_3S$: 472.1438; found: 472.1436.

Ethyl 8-(((1H-imidazol-4-yl)methyl)amino)-3-(4-methylbenzamido)thieno[3,2-c]quinoline-2-carboxylate (**2b**)

Yield 42%. Mp 150–152 °C. ^{1}H NMR (DMSO-d_6) δ: 1.25 (t, 3H, J = 7.1 Hz, CH_3), 2.42 (s, 3H, CH_3), 4.26–4.36 (m, 4H, 2×CH_2), 6.77 (t, 1H, J = 5.4 Hz, NH), 7.02 (d, 1H, J = 2.5 Hz, H-9), 7.07 (d, 1H, H-5″), 7.33 (dd, 1H, J = 9.1, 2.5 Hz, H-7), 7.40 (d, 2H, J = 8.0 Hz, H-3′, H-5′), 7.63 (d, 1H, H-2″), 7.85 (d, 1H, J = 9.0 Hz, H-6), 8.00 (d, 2H, J = 8 Hz , H-2′, H-6′), 8.84 (s, 1H, H-4), 10.60 (s, 1H, NH), 12.00 (br s, 1H, NH). ^{13}C NMR (DMSO-d_6) δ: 14.5 (CH_3), 21.6 (CH_3), 39.5 (CH_2), 61.9 (CH_2), 98.4 (CH), 121.1, 121.6 (CH), 124.8, 128.4 (CH), 129.6 (CH), 130.9 (CH), 131.1, 135.5 (CH), 137.7, 141.7, 142.0 (CH), 142.9, 148.6, 162.1, 165.9. HRMS-ESI [$(M+H)^+$]: m/z calculated for $C_{26}H_{23}N_5O_3S$: 486.1594; found: 486.1595.

Ethyl 8-(((1H-imidazol-4-yl)methyl)amino)-3-(4-methoxybenzoylamino)thieno[3,2-c]quinoline-2-carboxylate (**2c**)

Yield 48%. Mp 154–155 °C. ^{1}H NMR (DMSO-d_6) δ: 1.25 (t, 3H J = 7.1 Hz, CH_3), 3.87 (s, 3H, OCH_3), 4.26–4.36 (m, 4H, 2×CH_2), 6.76 (t, 1H, J = 5.4 Hz, NH), 7.02 (d, 1H, J = 2.5 Hz, H-9), 7.07 (s, 1H, H-5″), 7.13 (d, 2H, J = 8.8 Hz, H-3′, H-5′), 7.33 (dd, 1H, J = 9.1, 2.5 Hz, H-7), 7.62 (s, 1H, H-2″), 7.85 (d, J = 9.0 Hz, 1H, H-6), 8.08 (d, 2H, J = 8.7 Hz, H-2′, H-6′), 8.84 (s, 1H, H-4), 10.54 (s, 1H, NH), 11.97 (br s, 1H, NH). ^{13}C NMR (DMSO-d_6) δ: 14.5 (CH_3), 39.5 (CH_2), 56.0 (CH_3), 61.9 (CH_2), 98.4 (CH), 114.4 (CH), 120.7, 121.6 (CH), 124.8, 126.0, 129.6, 130.4 (CH), 130.9 (CH), 135.5 (CH), 138.0, 141.7, 142.1 (CH), 148.6, 162.1, 162.9, 165.4. HRMS-ESI [$(M+H)^+$]: m/z calculated for $C_{26}H_{23}N_5O_4S$: 502.1544; found: 502.1542.

Ethyl 8-(((1H-imidazol-4-yl)methyl)amino)-3-(4-trifluoromethylbenzoylamino)thieno[3,2-c]quinoline -2-carboxylate (**2d)**

Yield 28%. Mp 192-195 °C. ^{1}H NMR (400 MHz, DMSO-d_6) δ: 1.25 (t, 3H, J = 7.1 Hz, CH_3), 4.27 – 4.36 (m, 4H, 2 x CH_2), 6.77 (t, 1H, J = 5.4 Hz, NH), 7.04 (d, 1H, J = 2.5 Hz, H-9), 7.07 (s, 1H, H-5″), 7.34 (dd, 1H, J = 9.1, 2.5 Hz, H-7), 7.62 (s, 1H, H-2″), 7.86 (d, 1H, J = 9.1 Hz, H-6), 7.99 (d, 2H, J = 8.3 Hz, H-3′, H-5′), 8.28 (d, 2H, J = 8.1 Hz, H-2′, H-6′), 8.87 (s, 1H, H-4), 10.93 (s, 1H, NH), 11.96 (s, 1H, NH). ^{13}C NMR (DMSO-d_6) δ: 14.5 (CH_3), 39.5 (CH_2), 61.9 (CH_2),

98.4 (CH), 121.7 (CH), 122.3, 124.8, 126.1, 126.1, 129.3 (CH), 129.7, 131.0 (CH), 132.5, 135.5 (CH), 136.8, 137.9, 138.1, 141.6 (CH), 148.6, 161.7, 165.2. HRMS-ESI [(M+H)$^+$]: m/z calculated for $C_{26}H_{20}F_3N_5O_3S$: 540.1312; found: 540.1313.

Ethyl 8-(((1H-imidazol-4-yl)methyl)amino)-3-(3-chloro-4-fluorobenzamido)thieno[3,2-c]quinoline-2-carboxylate (**2e**)

Yield 37%. Mp 207–210 °C. ^{1}H NMR (DMSO-d$_6$) δ: 1.26 (t, 3H, J = 7.1 Hz, CH_3), 4.27–4.36 (m, 4H, 2×CH_2), 6.75 (t, 1H, J = 5.4 Hz, NH), 7.03 (d, 1H, J = 2.5 Hz, H-9), 7.07 (s, 1H, H-5″), 7.34 (dd, 1H, J = 9.1, 2.5 Hz, H-7), 7.62 (s, 1H, H-2″), 7.66 (t, 1H, J = 8.9 Hz, H-5′), 7.85 (d, 1H, J = 9.1 Hz, H-6), 8.11 (ddd, 1H, J = 8.7, 4.7, 2.2 Hz, H-6′), 8.32 (dd, 1H, J = 7.1, 2.2 Hz, H-2′), 8.85 (s, 1H, H-4), 10.80 (s, 1H, NH), 11.91 (br s, 1H, NH). ^{13}C NMR (DMSO-d$_6$) δ: 14.5 (CH_3), 40.5 (CH_2), 61.9 (CH_2), 98.3, 117.8 (d, J = 21.5 Hz, CH), 120.3, 120.4, 121.7 (CH), 122.2, 124.7, 129.6, 129.7 (d, J = 8.3 Hz, CH), 130.9 (CH), 131.7, 135.5 (CH), 136.8, 138.1, 141.6 (CH), 141.6, 148.6, 159.9 (d, J = 253.1 Hz), 161.6, 164.0. HRMS-ESI [(M+H)$^+$]: m/z calculated for $C_{25}H_{19}ClFN_5O_3S$: 524.0954; found: 524.0954.

Ethyl 3-benzoylamino-8-(((5-methyl-1H-imidazol-4-yl)methyl)amino)thieno[3,2-c]quinoline-2-carboxylate (**2f**)

Yield 65%. Mp 148-149 °C. ^{1}H NMR (DMSO-d$_6$) δ: 1.25 (t, 3H, J = 7.1 Hz, CH_3), 2.25 (s, 3H, CH_3), 4.24–4.36 (m, 4H, 2×CH_2), 6.67 (t, 1H, J = 5.2 Hz, NH), 7.03 (d, 1H, J = 2.5 Hz, H-9), 7.33 (dd, 1H, J = 9.1, 2.5 Hz, H-7), 7.48 (s, 1H, H-2″), 7.56–7.71 (m, 3H, H-3′, H-4′, H-5′), 7.84 (d, 1H J = 9.0 Hz, H-6), 8.06–8.14 (m, 2H, H-2′, H-6′), 8.84 (s, 1H, H-4), 10.68 (s, 1H, NH), 11.75 (br s, 1H, NH). ^{13}C NMR (DMSO-d$_6$) δ: 14.5 (CH_3), 19.0 (CH_3), 39.5 (CH_2), 61.9 (CH_2), 98.1 (CH), 121.4, 121.8 (CH), 124.8, 128.4 (CH), 129.1 (CH), 129.6, 130.9 (CH), 132.7 (CH), 133.7 (CH), 134.0, 137.5, 138.1, 141.6, 141.8 (CH), 148.6, 162.0, 166.1. HRMS-ESI [(M+H)$^+$]: m/z calculated for $C_{26}H_{23}N_5O_3S$: 486.1954; found: 486.1955.

Ethyl 8-(((5-methyl-1H-imidazol-4-yl)methyl)amino)-3-(4-methylbenzoylamino)thieno[3,2-c]quinoline-2-carboxylate (**2g**)

Yield 33%. Mp 157–159 °C. ^{1}H NMR (DMSO-d$_6$) δ: 1.25 (t, 3H, J = 7.1 Hz, CH_3), 2.30 (s, 3H, CH_3), 2.42 (s, 3H, CH_3), 4.26–4.36 (m, 4H, 2×CH_2), 6.72 (t, 1H, J = 5.2 Hz, NH), 7.03 (d, 1H, J = 2.4 Hz, H-9), 7.31 (dd, 1H, J = 9.1, 2.5 Hz, H-7), 7.40 (d, 2H, J = 7.9 Hz, H-3′, H-5′), 7.86 (d, 1H, J = 9.1 Hz, H-6), 7.95–8.03 (m, 3H, H-2′, H-6′, H-2″), 8.86 (s, 1H, H-4), 10.60 (s, 1H, NH), 11.90 (br s, 1H, NH). ^{13}C NMR (DMSO-d$_6$): 10.2 (CH_3), 14.5 (CH_3), 21.6 (CH_3), 38.7 (CH_2), 61.9 (CH_2), 98.4 (CH), 121.1, 121.7 (CH), 124.8, 126.1, 128.4 (CH), 129.2, 129.6 (CH), 131.0 (CH), 131.1, 133.6 (CH), 137.7, 138.2, 141.7, 142.2 (CH), 142.9, 148.3, 162.1, 165.9. HRMS-ESI [(M+H)$^+$]: m/z calculated for $C_{27}H_{25}N_5O_3S$: 500.1751; found: 500.1750.

Ethyl 8-(((5-methyl-1H-imidazol-4-yl)methyl)amino)-3-(4-methoxybenzoylamino)thieno[3,2-c] quinoline-2-carboxylate (**2h**)

Yield 51%. Mp 200–202 °C. ^{1}H NMR (DMSO-d$_6$) δ: 1.25 (t, 3H, J = 7.1 Hz, CH_3), 2.25 (s, 3H, CH_3), 3.87 (s, 3H, OCH_3), 4.23–4.36 (m, 4H, 2×CH_2), 6.68 (t, 1H, J = 5.1 Hz, NH), 7.02 (d, 1H, J = 2.6 Hz, H-9), 7.13 (d, 2H, J = 8.9 Hz, H-3′, H-5′), 7.32 (dd, 1H, J = 9.1, 2.5 Hz, H-7), 7.56 (s, 1H, H-2″), 7.83 (d, 1H, J = 9.1 Hz, H-6), 8.08 (d, 2H, J = 8.8 Hz, H-2′, H-6′), 8.83 (s, 1H, H-4),

10.54 (s, 1H, NH), 11.97 (br s, 1H, NH). ^{13}C NMR (DMSO-d_6) δ: 10.5 (CH_3), 14.5 (CH_3), 39.5 (CH_2), 56.0 (CH_3), 61.9 (CH_2), 98.2 (CH), 114.4 (CH), 120.6, 121.7 (CH), 124.8, 126.1, 129.6, 130.4 (CH), 130.9 (CH), 133.7 (CH), 138.0, 141.7, 142.1 (CH), 148.6, 162.2, 162.9, 165.4. HRMS-ESI [$(M+H)^+$]: m/z calculated for $C_{27}H_{25}N_5O_4S$: 516.1700; found: 516.1700.

Ethyl 8-(((5-methyl-1H-imidazol-4-yl)methyl)amino)-3-(4-(trifluoromethyl)benzamido)thieno [3,2-c]quinoline-2-carboxylate (**2i**)

Yield 30%. Mp 212–214 °C. ^1H NMR (400 MHz, DMSO-d_6) δ: 1.25 (t, 3H, J = 7.1 Hz, CH_3), 2.25 (s, 3H, CH_3), 4.22 – 4.36 (m, 4H, 2 x CH_2), 6.67 (t, 1H, J = 5.1 Hz, NH), 7.04 (d, 1H, J = 2.5 Hz, H-9), 7.33 (dd, 1H, J = 9.1, 2.5 Hz, H-7), 7.48 (s, 1H, H-2′′), 7.84 (d, 1H, J = 9.0 Hz, H-6), 7.99 (d, 2H, J = 8.2 Hz, H-2′, H-6′), 8.28 (d, 2H, J = 8.1 Hz, H-3′, H-5′), 8.87 (s, 1H, H-4), 10.94 (s, 1H, NH), 11.77 (s, 1H, NH). ^{13}C NMR (DMSO-d_6) δ: 10.5 (CH_3), 14.5 (CH_3), 39.5 (CH_2), 61.9 (CH_2), 98.1 (CH), 121.8 (CH), 124.8, 126.1 (CH), 129.3 (CH), 129.7, 130.9 (CH), 133.7 (CH), 136.8, 138.0, 141.5 (CH), 141.6, 148.6, 161.8, 165.2. HRMS-ESI [$(M+H)^+$]: m/z calculated for $C_{27}H_{22}F_3N_5O_3S$: 554.1468; found: 554.1470.

Ethyl 3-(3-chloro-4-fluorobenzamido)-8-(((5-methyl-1H-imidazol-4-yl)methyl)amino)thieno[3,2-c]quinoline-2-carboxylate (**2j**)

Yield 32%. Mp 149–152 °C. ^1H NMR (DMSO-d_6) δ: 1.25 (t, 3H, J = 7.1 Hz, CH_3), 2.26 (s, 3H, CH_3), 4.25–4.38 (m, 4H, 2×CH_2), 6.68 (t, 1H, J = 5.2 Hz, NH), 7.04 (d, 1H, J = 2.5 Hz, H-9), 7.32 (dd, 1H, J = 9.1, 2.5 Hz, H-7), 7.63 (s, 1H, H-2″), 7.67 (t, 1H, J = 8.9 Hz, H-5′), 7.85 (d, 1H, J = 9.0 Hz, H-6), 8.11 (ddd, 1H, J = 8.7, 4.7, 2.3 Hz, H-6′), 8.32 (dd, 1H, J = 7.1, 2.2 Hz, H-2′), 8.85 (s, 1H, H-4), 10.80 (s, 1H, NH), 12.01 (br s, 1H, NH). ^{13}C NMR (DMSO-d_6) δ: 10.4 (CH_3), 14.5 (CH_3), 39.2 (CH_2), 61.9 (CH_2), 98.1 (CH), 117.8 (d, *J* = 21.7 Hz, CH), 120.3, 120.5, 121.8 (CH), 122.3, 124.7, 129.6, 129.7 (d, *J* = 9.0 Hz, CH), 130.9 (CH), 131.7, 133.7 (CH), 136.7, 138.1, 141.6 (CH), 148.5, 159.9 (d, *J* = 253.1 Hz), 161.7, 164.0. HRMS-ESI [$(M+H)^+$]: m/z calculated for $C_{26}H_{21}ClFN_5O_3S$: 538.1110; found: 538.1109.

Supporting Figures

Figure S1. Concentration–response curves of the imidazole-functionalized thieno[3,2-c]quinolines **2a–j** in TT (RET^{C634R}) medullary thyroid carcinoma cells after 3 days (red) and 6 days (green) of treatment, determined by MTT assay. Compound concentrations are expressed as logarithmic molar concentrations (Log[M]). Cell viability is expressed as percentage relative to untreated control cells. Data were fitted according to the Hill equation. Data are reported as mean ± SD from two independent experiments, each performed in quadruplicate.

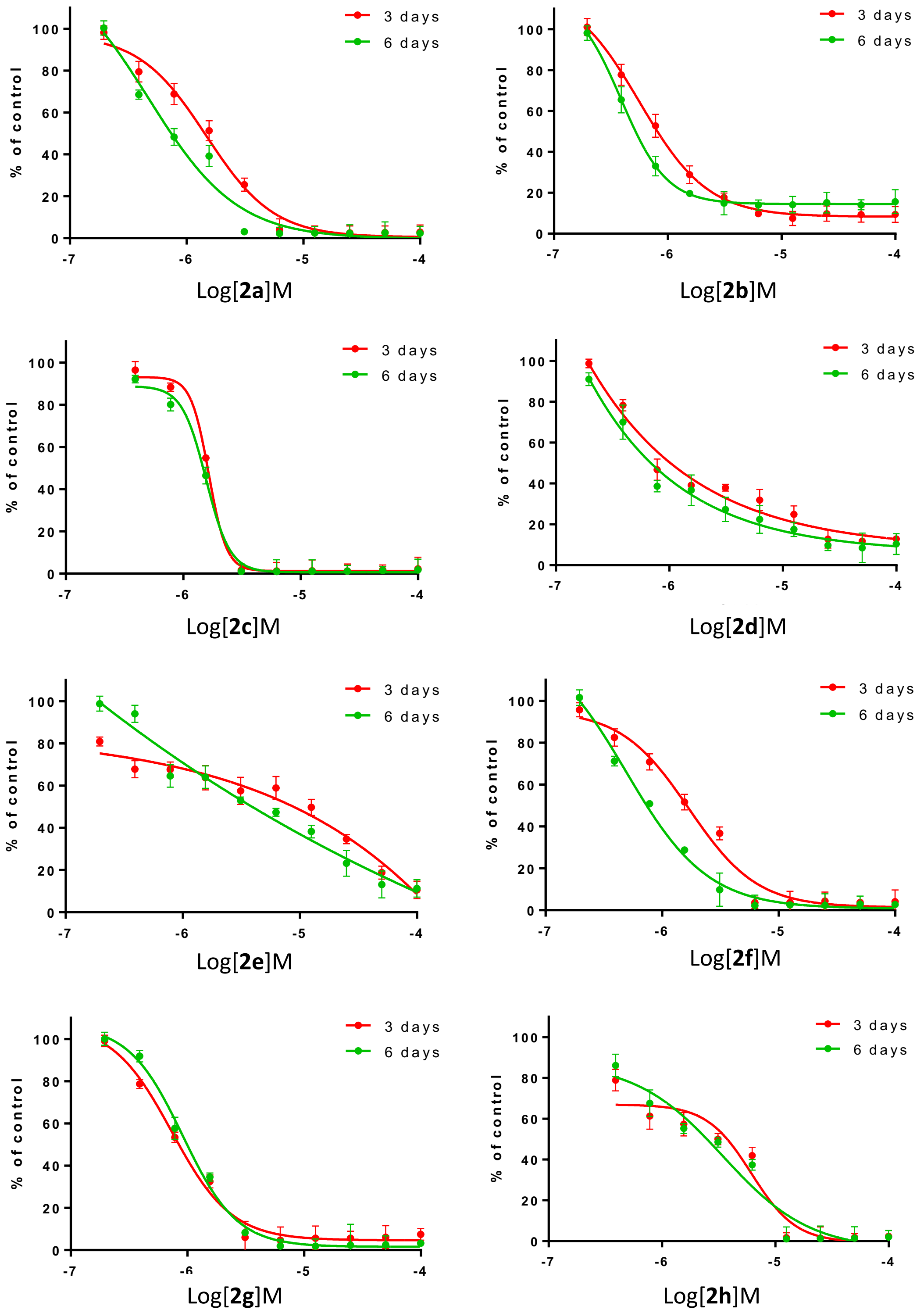

(Continued on next page)

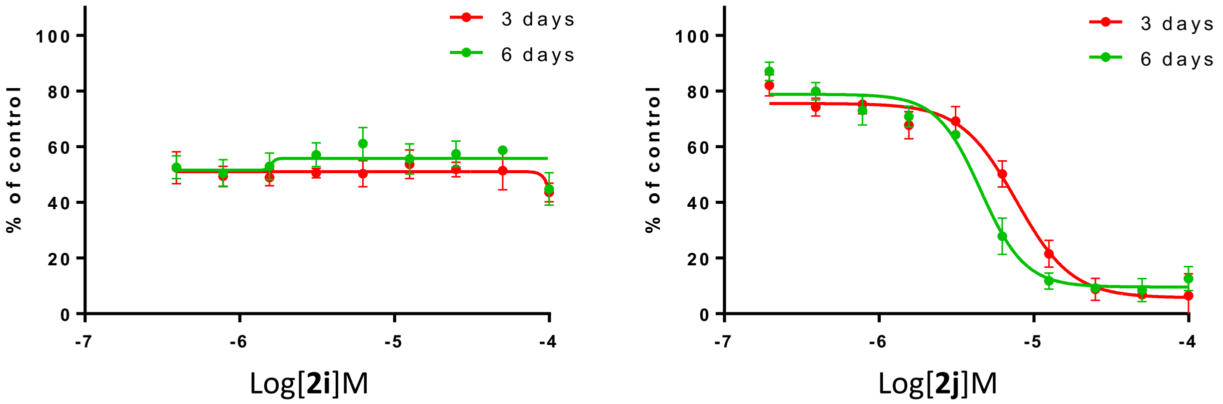

3 days
6 days
100
80
60
40
20
0
% of control
-7
-6
-5
-4
Log[2i]M
3 days
6 days
100
80
60
40
20
0
% of control
-7
-6
-5
-4
Log[2j]M

Figure S2. Concentration–response curves of the imidazole-functionalized thieno[3,2-c]quinolines **2a–j** in MZ-CRC-1 (RET^{M918T}) medullary thyroid carcinoma cells after 3 days (red) and 6 days (green) of treatment, determined by MTT assay. Compound concentrations are expressed as logarithmic molar concentrations (Log[M]). Cell viability is reported as percentage relative to untreated control cells. Data were fitted according to the Hill equation. Data are reported as mean ± SD from two independent experiments, each performed in quadruplicate.

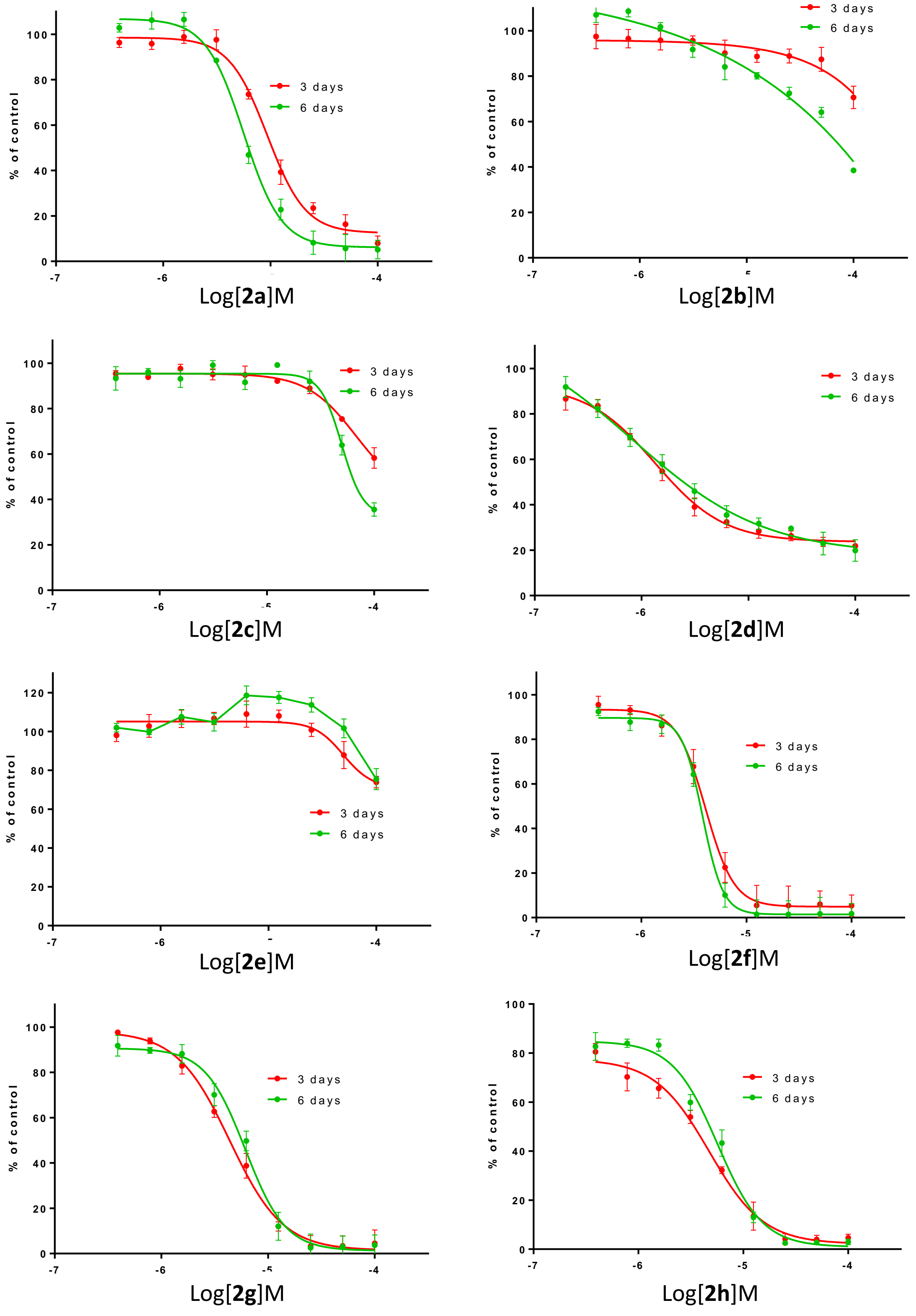

(Continued on next page)

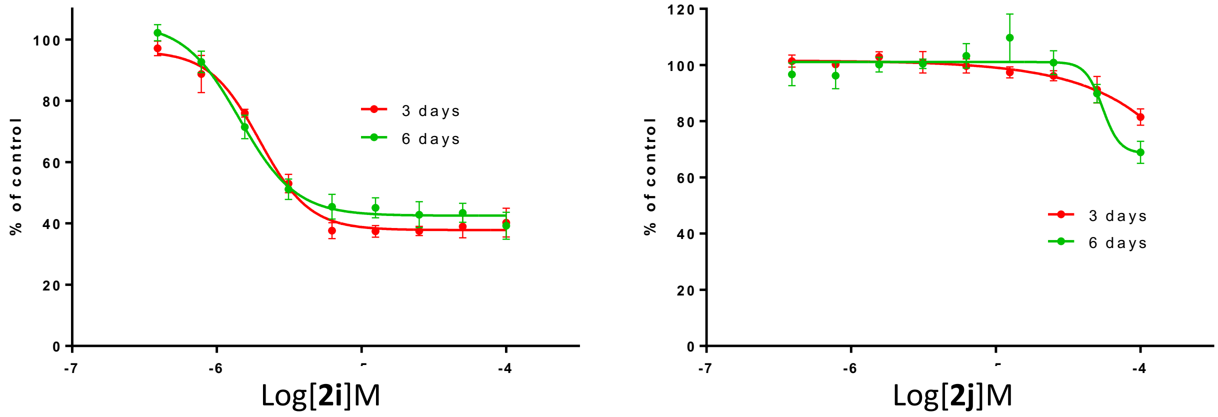
100
80
60
40
20
0
% of control
3 days
6 days
-7
-6
-4
Log[2i]M
120
100
80
60
40
20
0
% of control
3 days
6 days
-7
-6
-4
Log[2j]M

Figure S3. Root mean square deviation (RMSD) plots obtained from molecular dynamics simulations of the RET^{WT} kinase domain (PDB 6NEC) in the apo form and in complex with representative clinically relevant RET inhibitors, the co-crystallized ligand nintedanib and reference compound **1b**. Protein C*α* RMSD and ligand RMSD values were monitored throughout the simulation trajectories to evaluate the structural stability of the complexes and the persistence of ligand accommodation within the ATP-binding pocket.

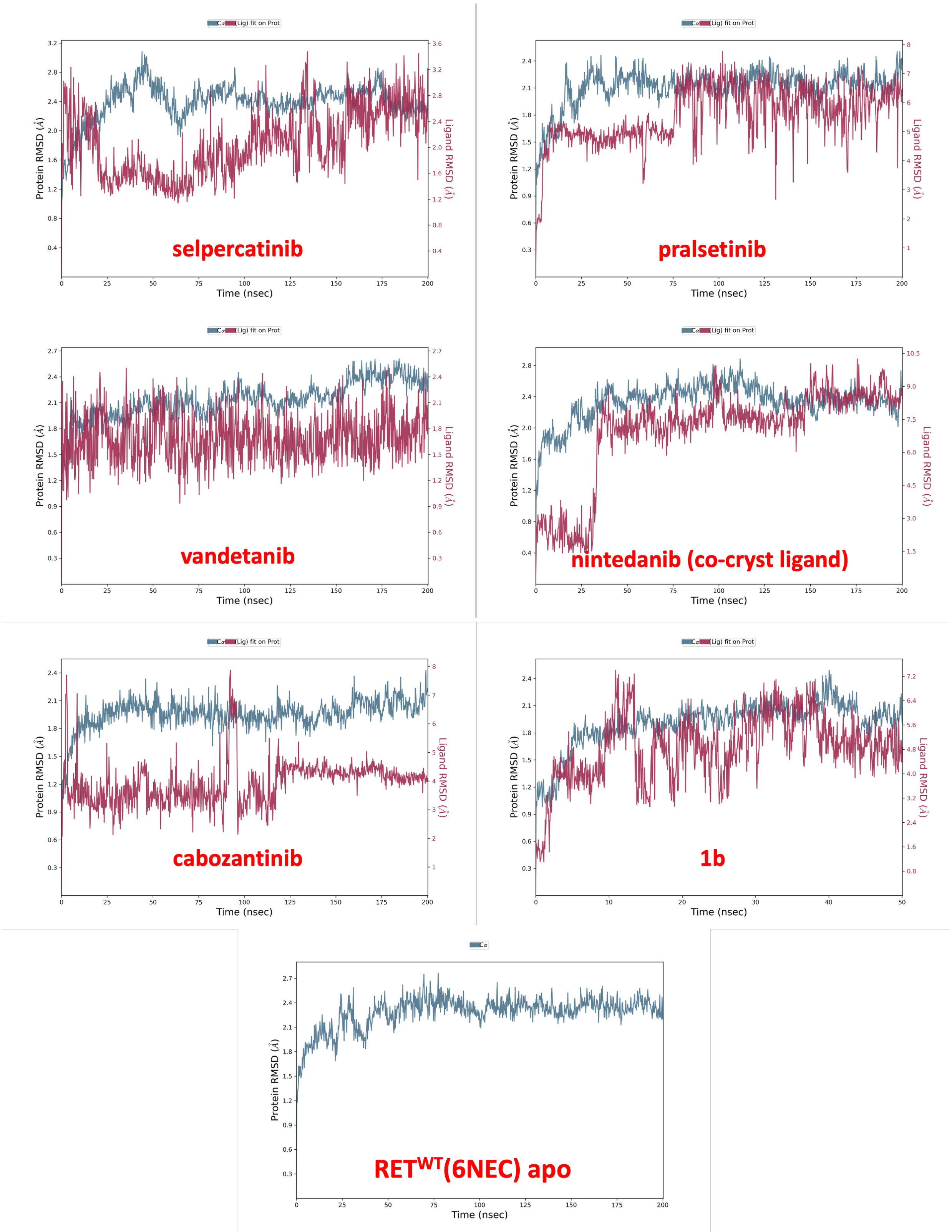

Figure S4. Root mean square fluctuation (RMSF) profiles of the RET^{WT} kinase domain (PDB 6NEC) during molecular dynamics simulations of the apo structure and complexes with representative clinically relevant RET inhibitors, the co-crystallized ligand nintedanib and reference compound **1b**. RMSF values were calculated for $C\alpha$ atoms over the simulation trajectories in order to evaluate residue flexibility and local conformational fluctuations within the kinase domain.

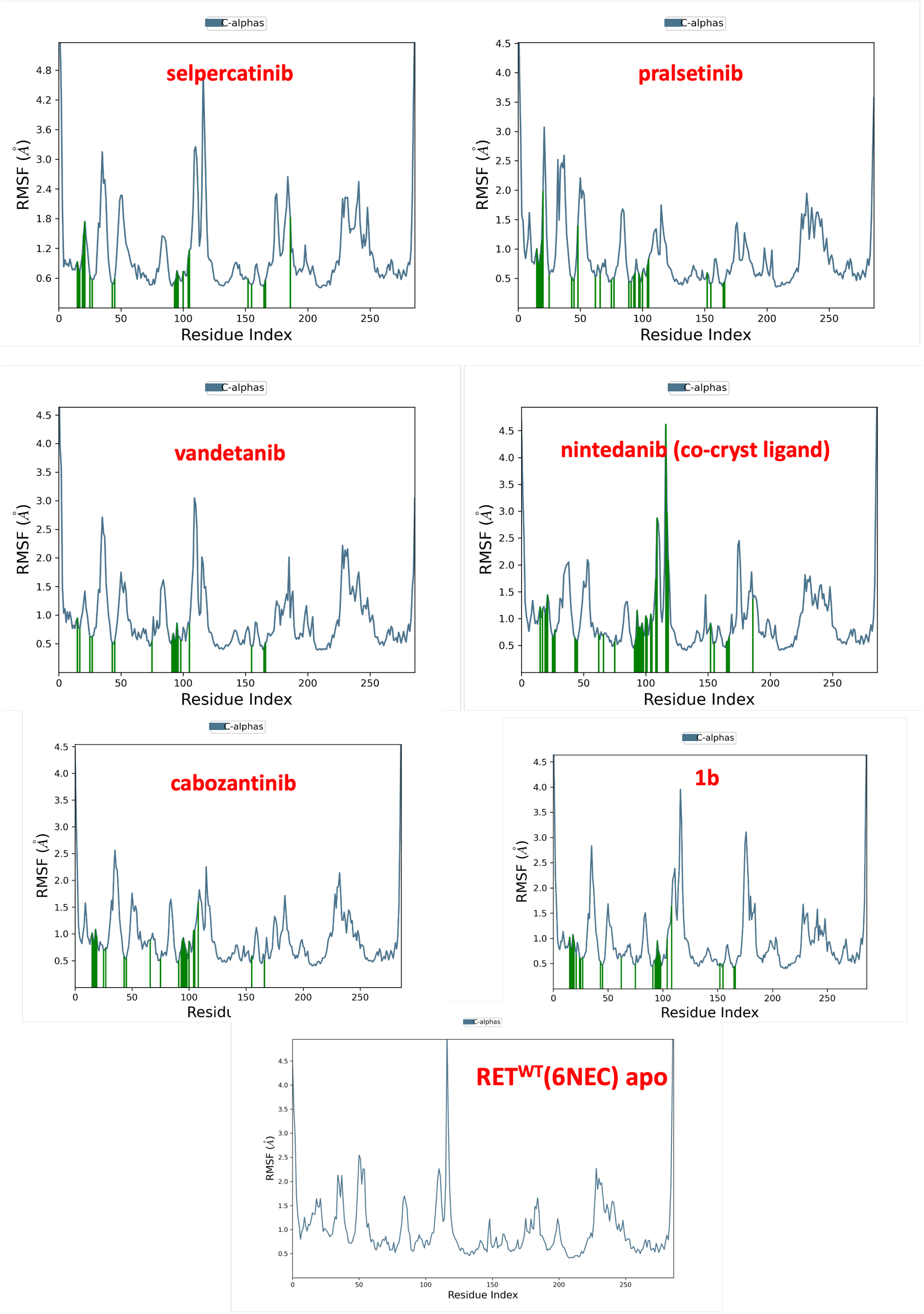

Ligand Contacts: Protein residues that interact with the ligand are marked with green-colored vertical bars.

Figure S5. Two-dimensional ligand–protein interaction diagrams of the best-ranked induced fit docking poses obtained for representative clinically relevant RET inhibitors, the co-crystallized ligand nintedanib and reference compound **1b**. The interaction maps highlight the main hydrogen-bonding, hydrophobic, water-mediated, and polar contacts established within the ATP-binding region of RET^{WT}.

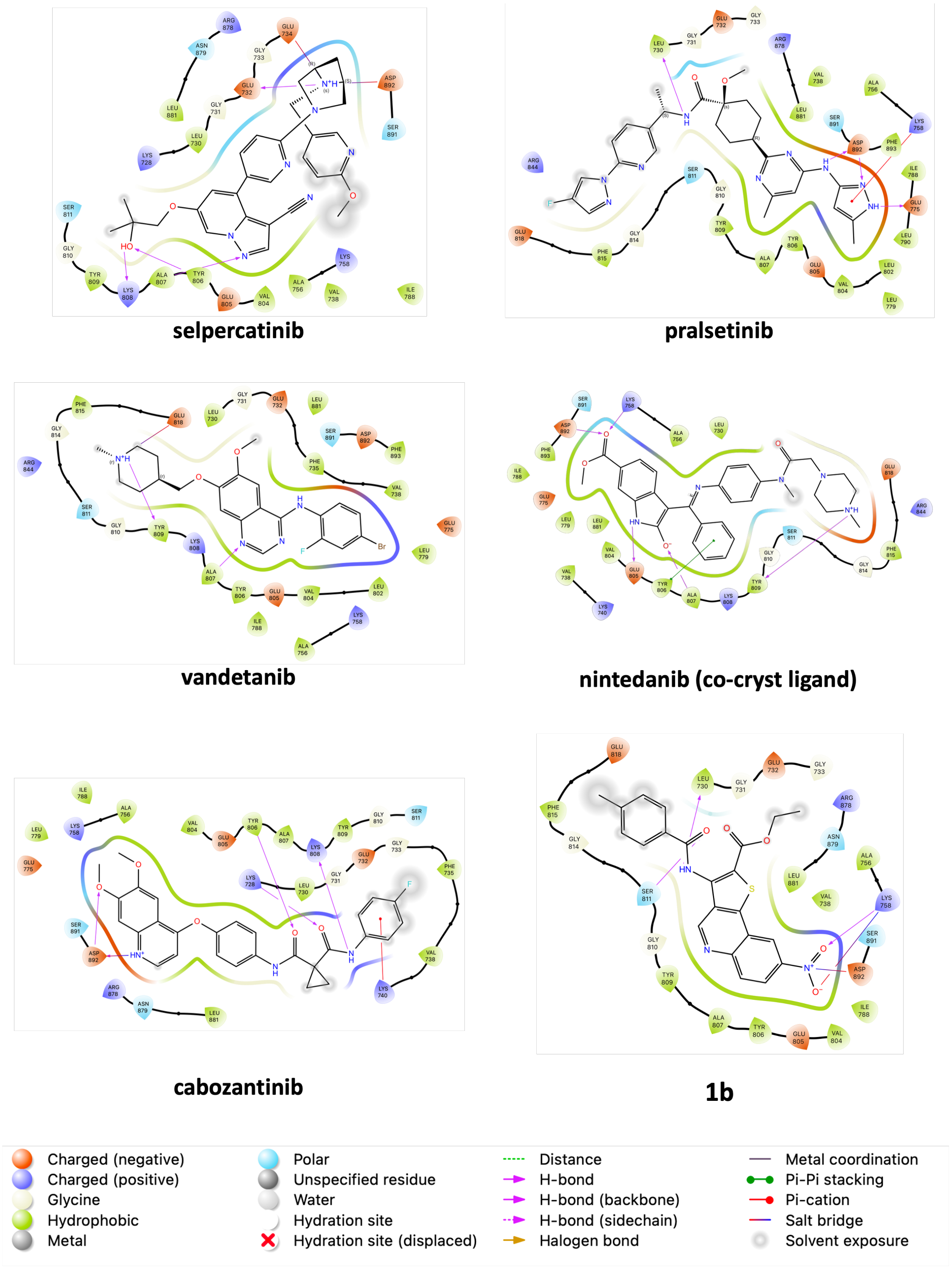

Figure S6. Protein–ligand interaction fraction analysis obtained from molecular dynamics simulations of the RET^{WT} kinase domain (PDB 6NEC) in complex with compounds **2b,g**, representative clinically relevant RET inhibitors, the co-crystallized ligand nintedanib and reference compound **1b**. Reported interaction fractions describe the persistence of hydrogen-bonding, hydrophobic, ionic, and water-bridge contacts established with key amino acid residues during the simulation trajectories.

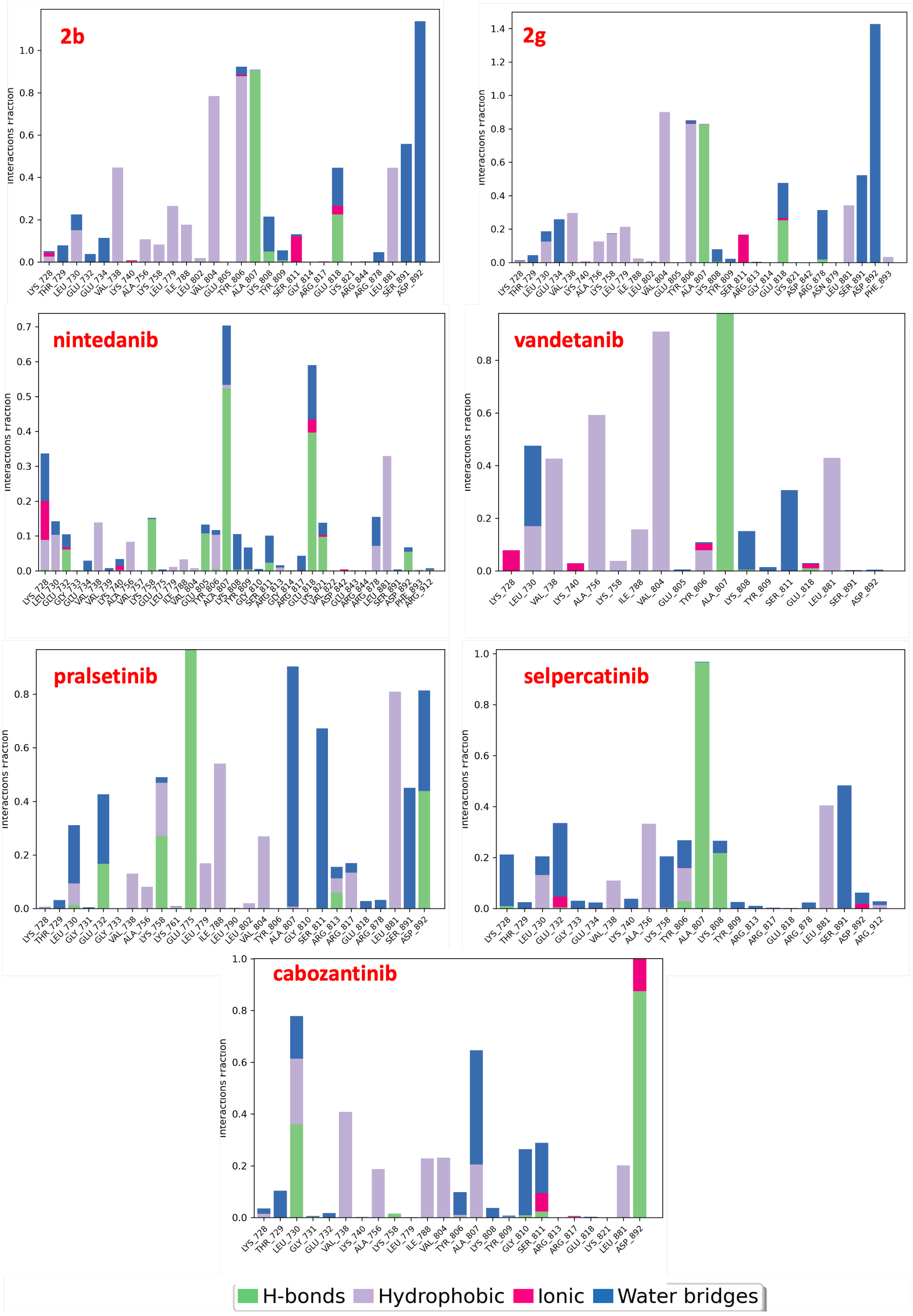

Figure S7. Root mean square deviation (RMSD) plots obtained from molecular dynamics simulations of the RET^{M918T} kinase domain (PDB 4CKI) in the apo form and in complex with representative clinically relevant RET inhibitors, the co-crystallized ligand adenosine and reference compound **1b**. Protein Cα RMSD and ligand RMSD values were monitored throughout the simulation trajectories to evaluate the structural stability of the complexes and the persistence of ligand accommodation within the ATP-binding pocket.

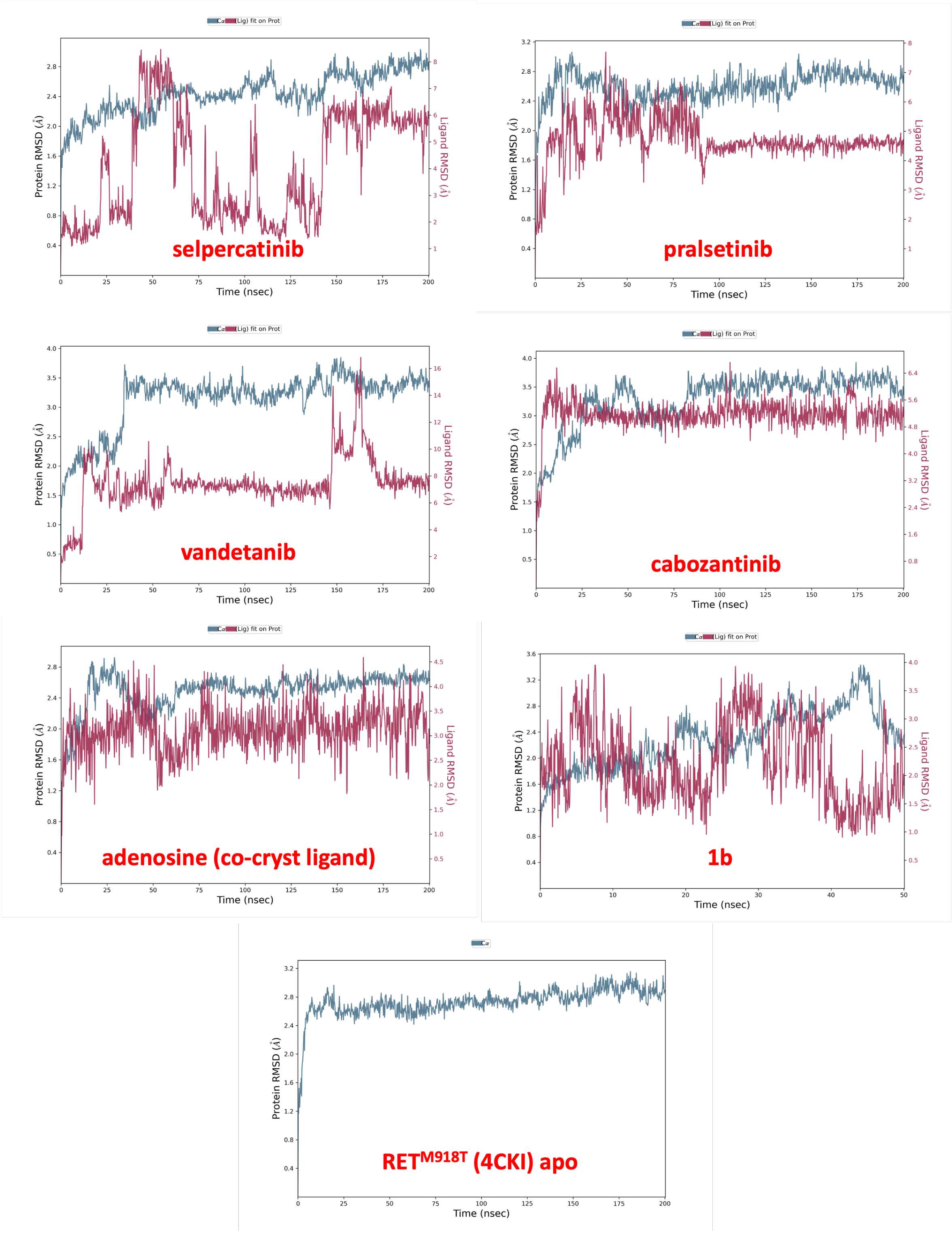

Figure S8. Root mean square fluctuation (RMSF) profiles of the RET^{M918T} kinase domain (PDB 4CKI) during molecular dynamics simulations of the apo structure and complexes with representative clinically relevant RET inhibitors, the co-crystallized ligand adenosine and reference compound **1b**. RMSF values were calculated for Cα atoms over the simulation trajectories in order to evaluate residue flexibility and local conformational fluctuations within the kinase domain.

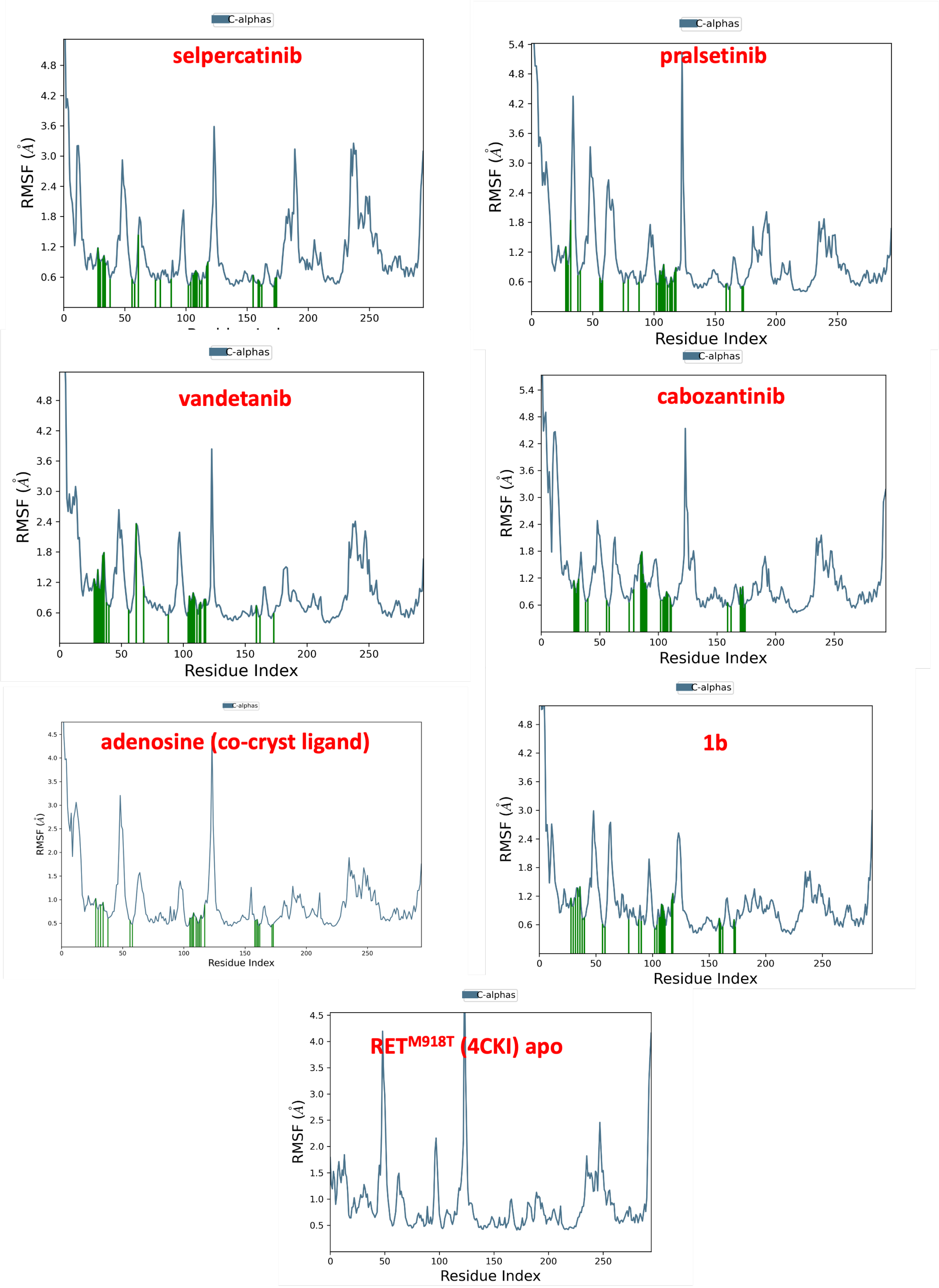

Ligand Contacts: Protein residues that interact with the ligand are marked with green-colored vertical bars.

Figure S9. Two-dimensional ligand–protein interaction diagrams of the best-ranked induced fit docking poses obtained for representative clinically relevant RET inhibitors, the co-crystallized ligand adenosine and reference compound **1b**. The interaction maps highlight the main hydrogen-bonding, hydrophobic, water-mediated, and polar contacts established within the ATP-binding region of RET^{M918T}.

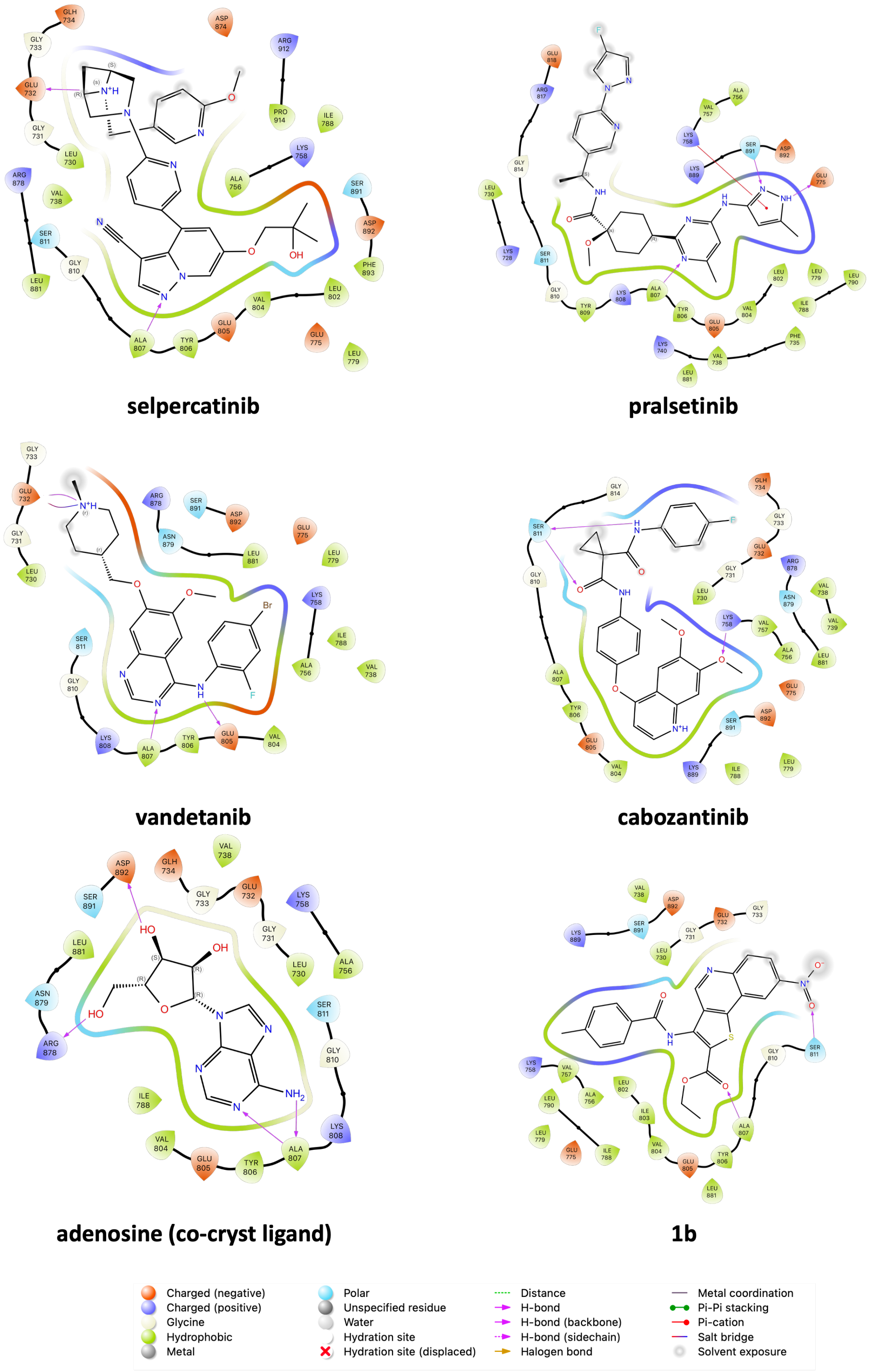

Figure S10. Protein–ligand interaction fraction analysis obtained from molecular dynamics simulations of the RET^{M918T} kinase domain (PDB 4CKI) in complex with compounds **2d,i**, representative clinically relevant RET inhibitors, the co-crystallized ligand adenosine and reference compound **1b**. Reported interaction fractions describe the persistence of hydrogen-bonding, hydrophobic, ionic, and water-bridge contacts established with key amino acid residues during the simulation trajectories.

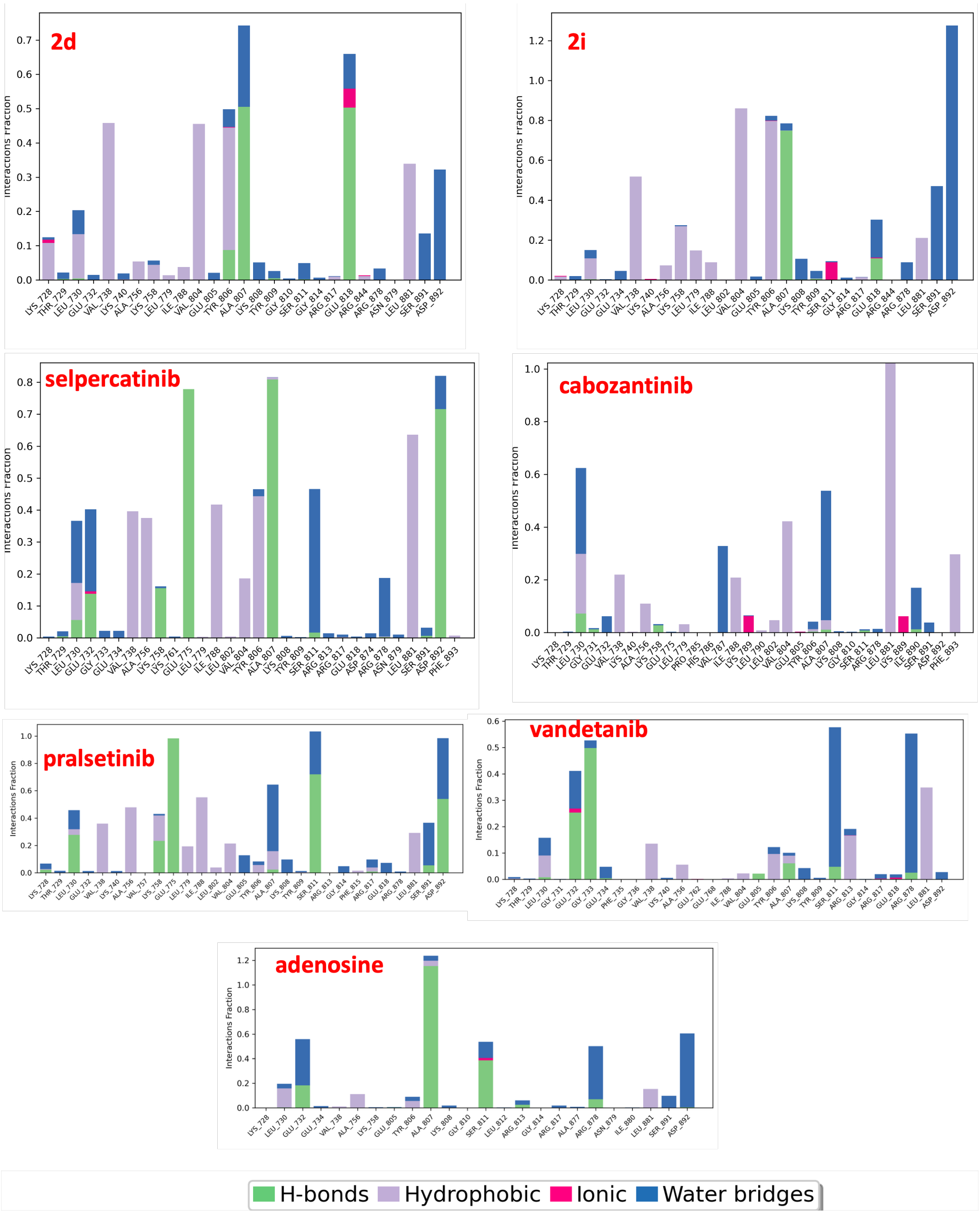

Figure S11. Root mean square deviation (RMSD) plots obtained from molecular dynamics simulations of the PI3Kα catalytic domain (PDB 8EXL) in the apo form and in complex with representative clinically relevant PI3Ks inhibitors, the co-crystallized ligand taselisib and reference compound **1b**. Protein Cα RMSD and ligand RMSD values were monitored throughout the simulation trajectories to evaluate the structural stability of the complexes and the persistence of ligand accommodation within the ATP-binding pocket.

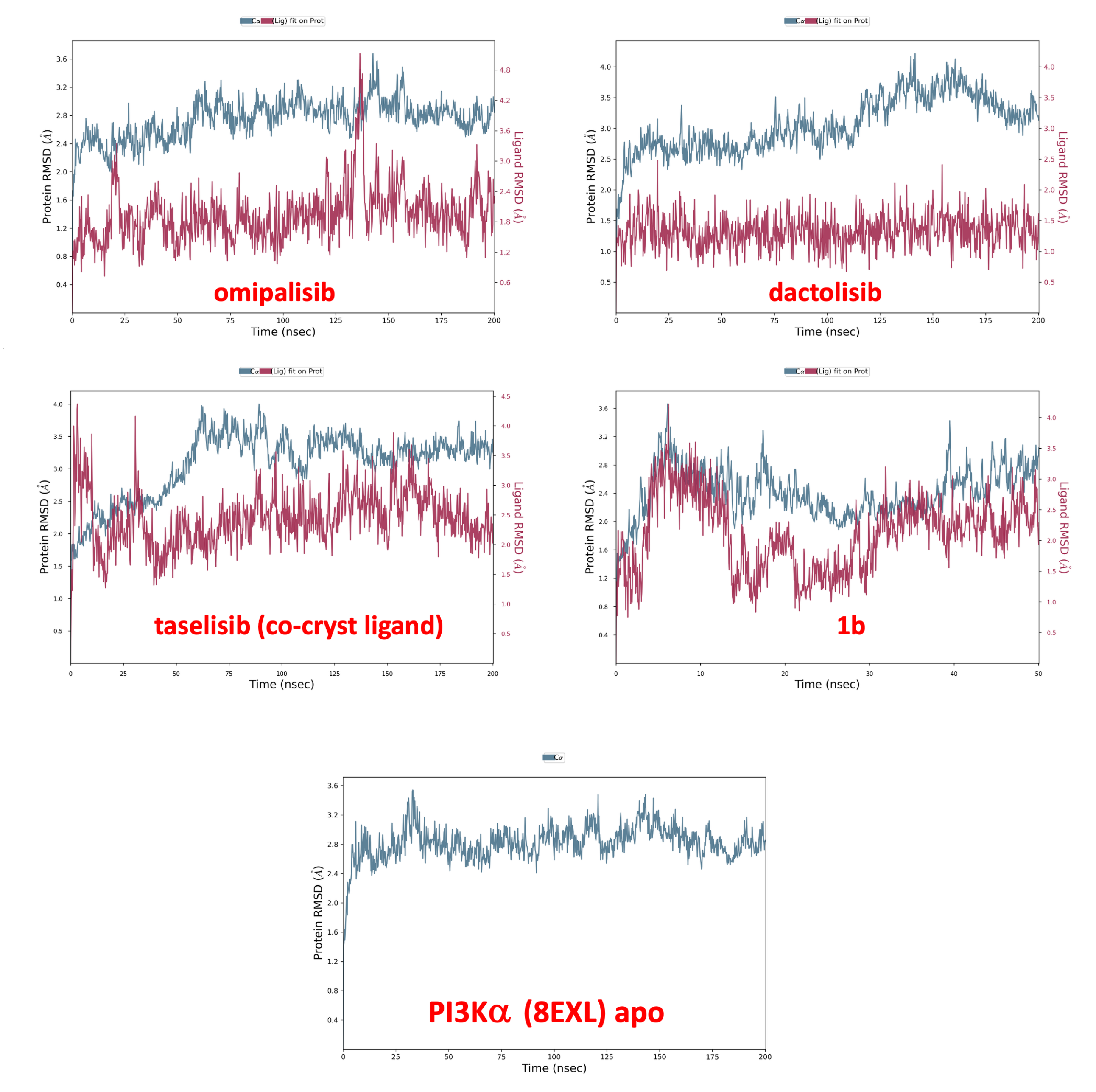

Figure S12. Root mean square fluctuation (RMSF) profiles of the PI3Kα catalytic domain (PDB 8EXL) in the apo form and in complex with representative clinically relevant PI3Ks inhibitors, the co-crystallized ligand taselisib and reference compound **1b**. RMSF values were calculated for C*α* atoms over the simulation trajectories in order to evaluate residue flexibility and local conformational fluctuations within the kinase domain.

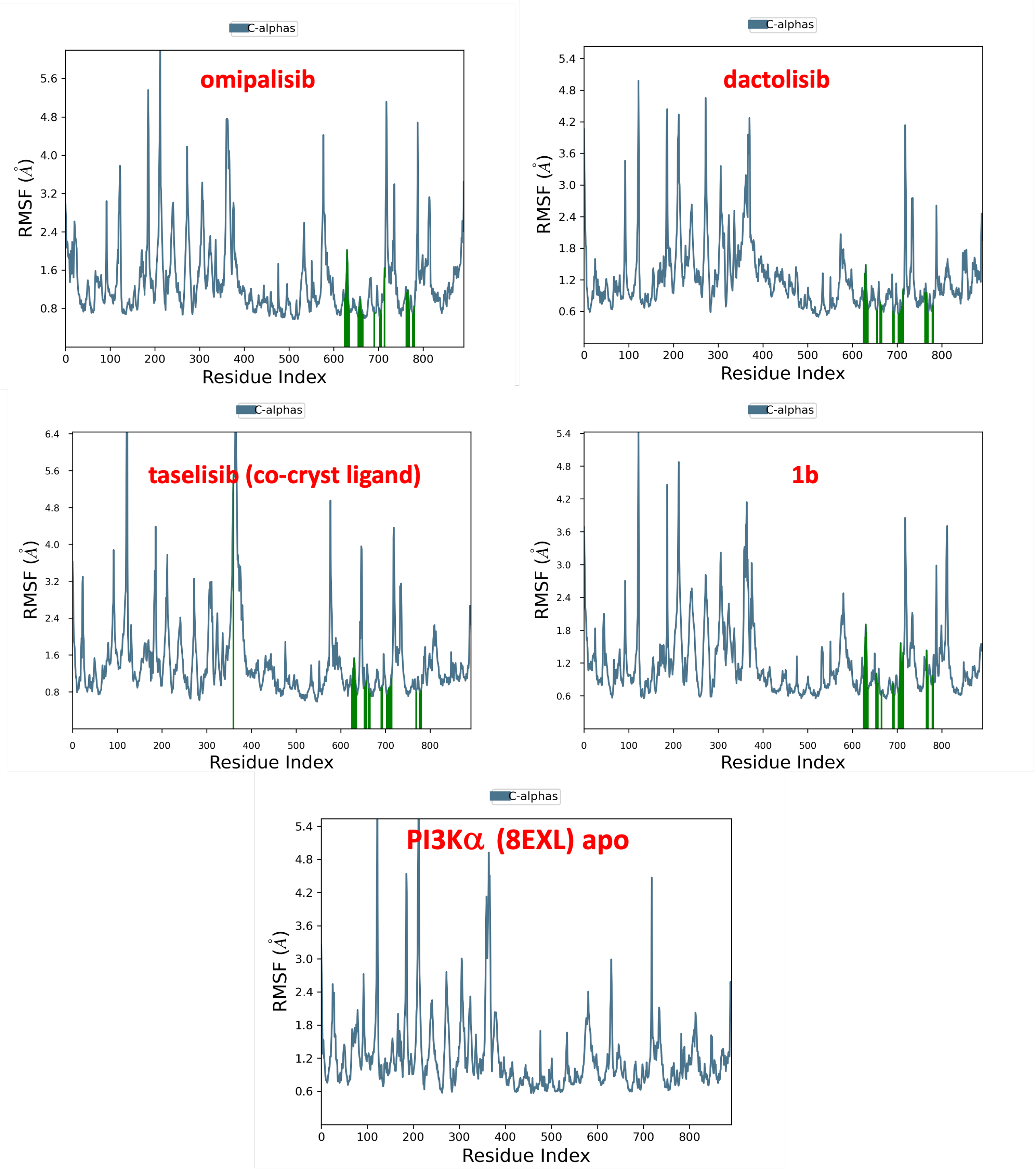

Figure S13. Two-dimensional ligand–protein interaction diagrams of the best-ranked induced fit docking poses obtained for representative clinically relevant PI3K inhibitors, the co-crystallized ligand taselisib and reference compound **1b**. The interaction maps highlight the main hydrogen-bonding, hydrophobic, water-mediated, and polar contacts established within the PI3Kα catalytic domain.

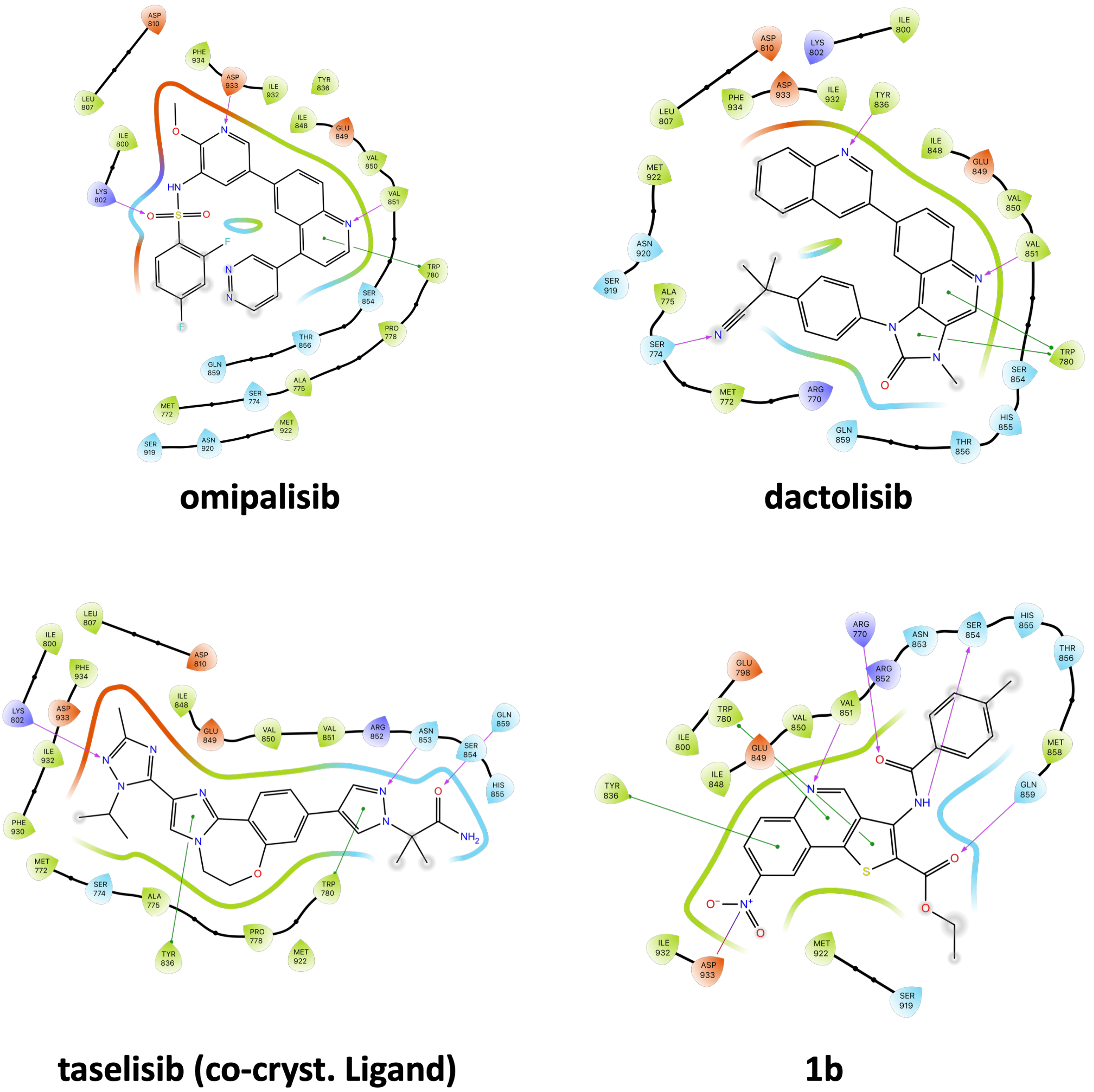

Figure S14. Protein–ligand interaction fraction analysis obtained from molecular dynamics simulations of the PI3Kα catalytic domain (PDB 8EXL) in complex with compounds **2b,g**, representative clinically relevant PI3Ks inhibitors, the co-crystallized ligand taselisib and reference compound **1b**. Reported interaction fractions describe the persistence of hydrogen-bonding, hydrophobic, ionic, and water-bridge contacts established with key amino acid residues during the simulation trajectories.

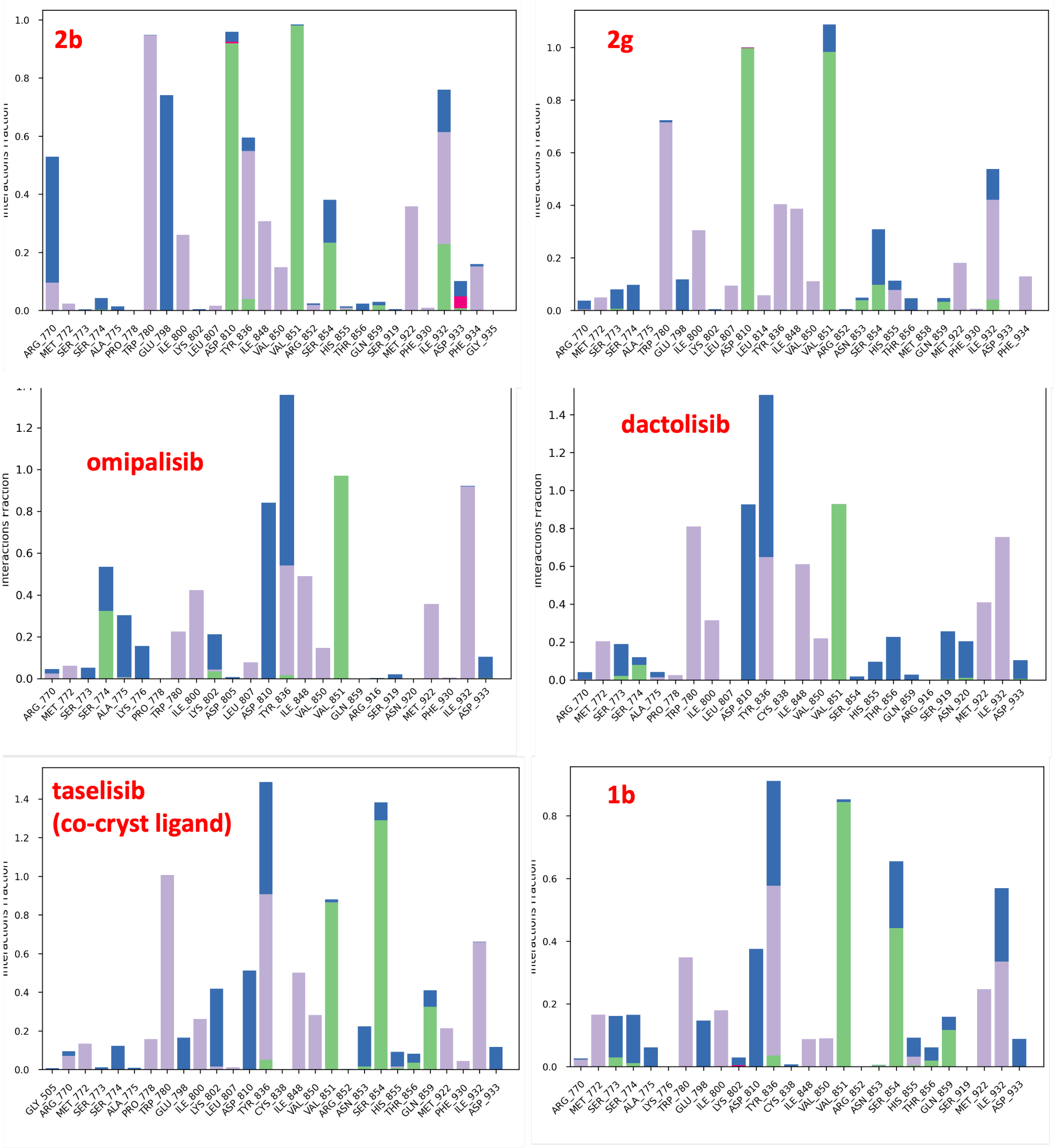

Figure S15. Predicted toxicity and off-target interaction profiles of compounds **2b,d,g,i** obtained using the ProTox-3.0 platform. Upper panels: radar plots summarizing the predicted probabilities associated with major toxicity endpoints and biological target classes in comparison with the average profile of active compounds within each category. Lower panels: predicted off-target interaction heatmaps for selected safety-relevant receptors and enzymes. Overall, the investigated compounds displayed no major toxicity alerts or significant predicted interactions with the analyzed off-targets, supporting their preliminary safety profile.

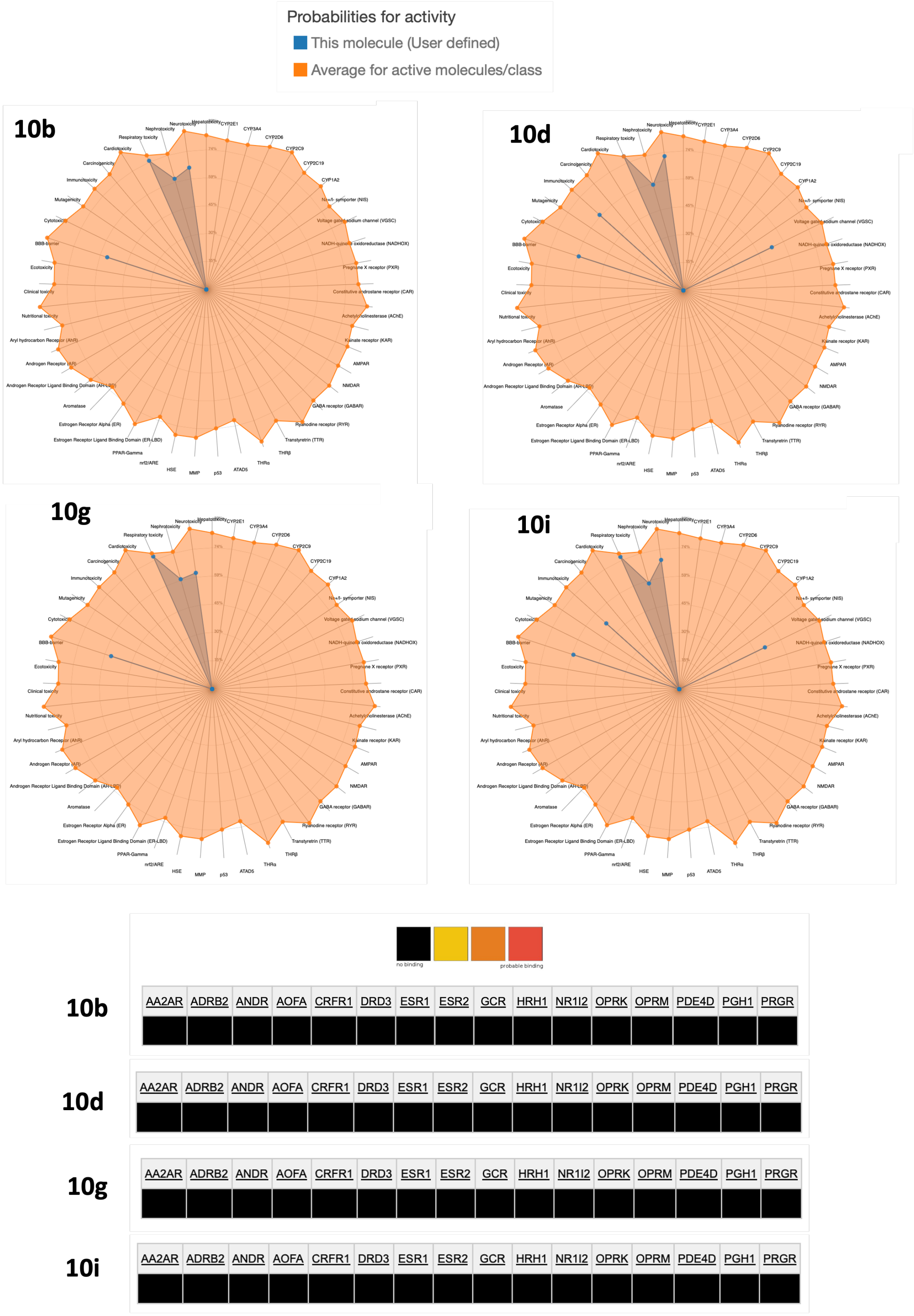

Figure S16. ^{1}HNMR spectrum of compound **2b** (400 MHz, d_6-DMSO)

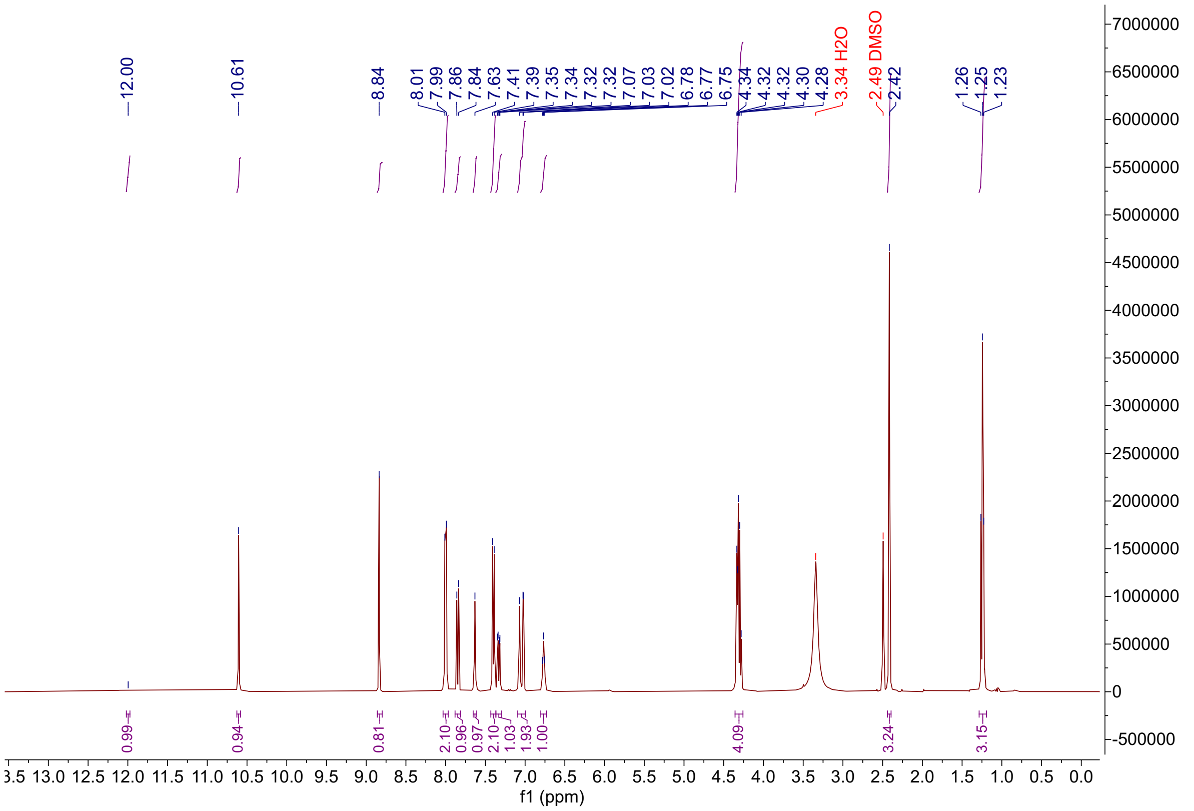

Figure S17. ^{13}CNMR spectrum of compound **2b** (100 MHz, d_6-DMSO)

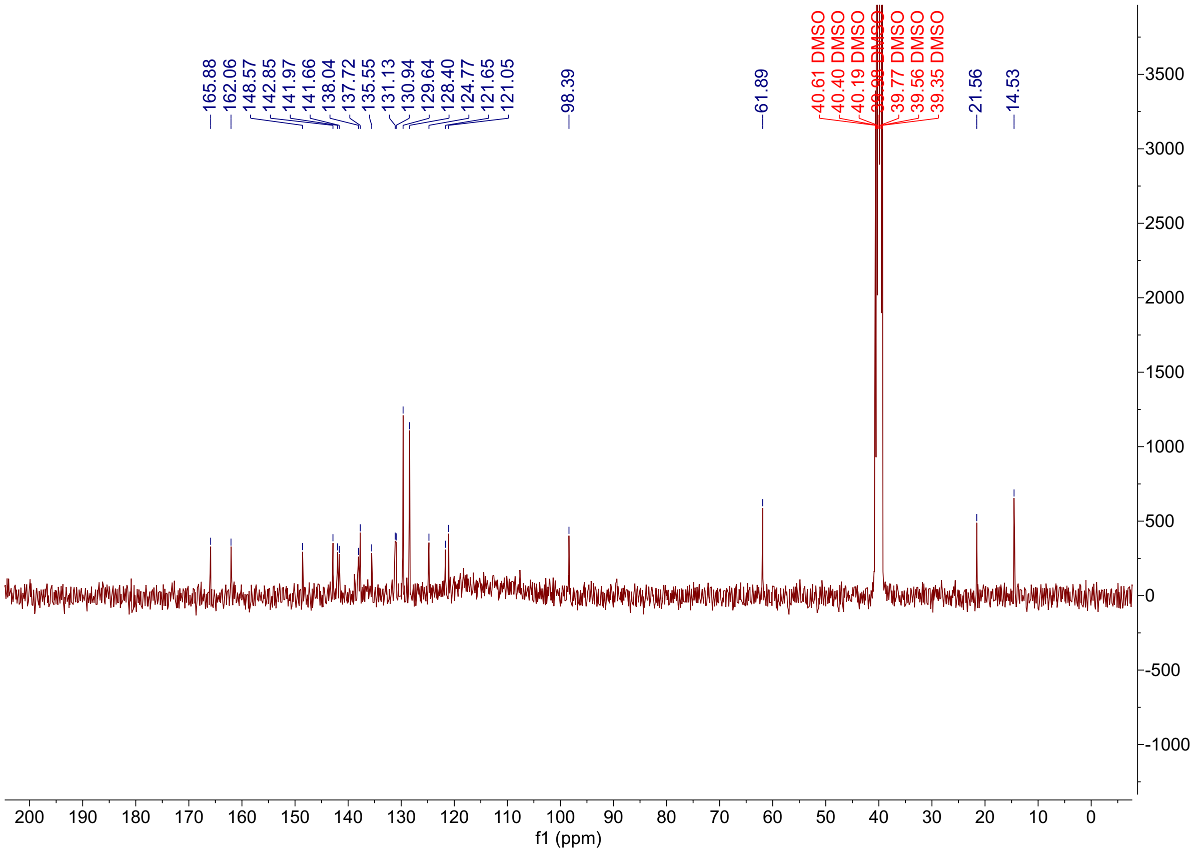

Figure S18: ^{1}HNMR spectrum of compound **2d** (400 MHz, d_6-DMSO)

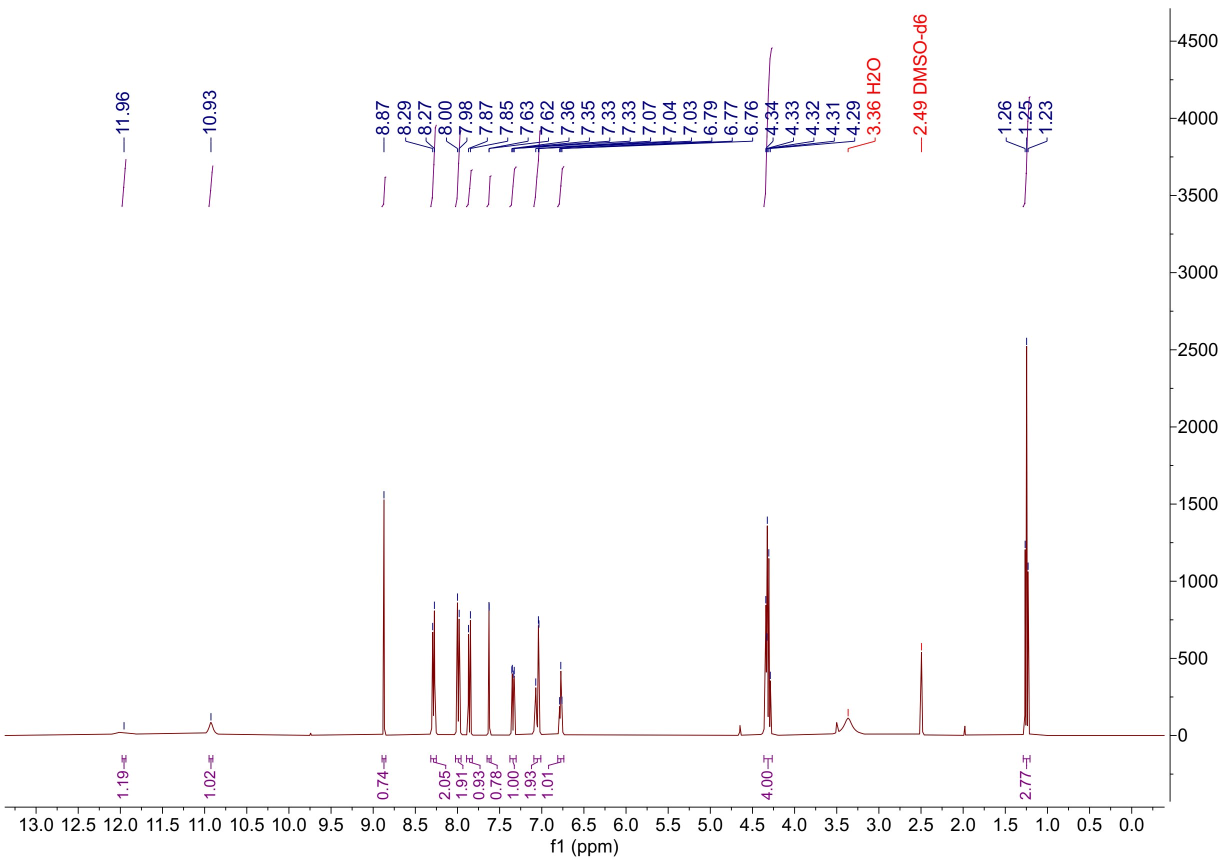

Figure S19. ^{13}CNMR spectrum of compound **2d** (100 MHz, d_6-DMSO)

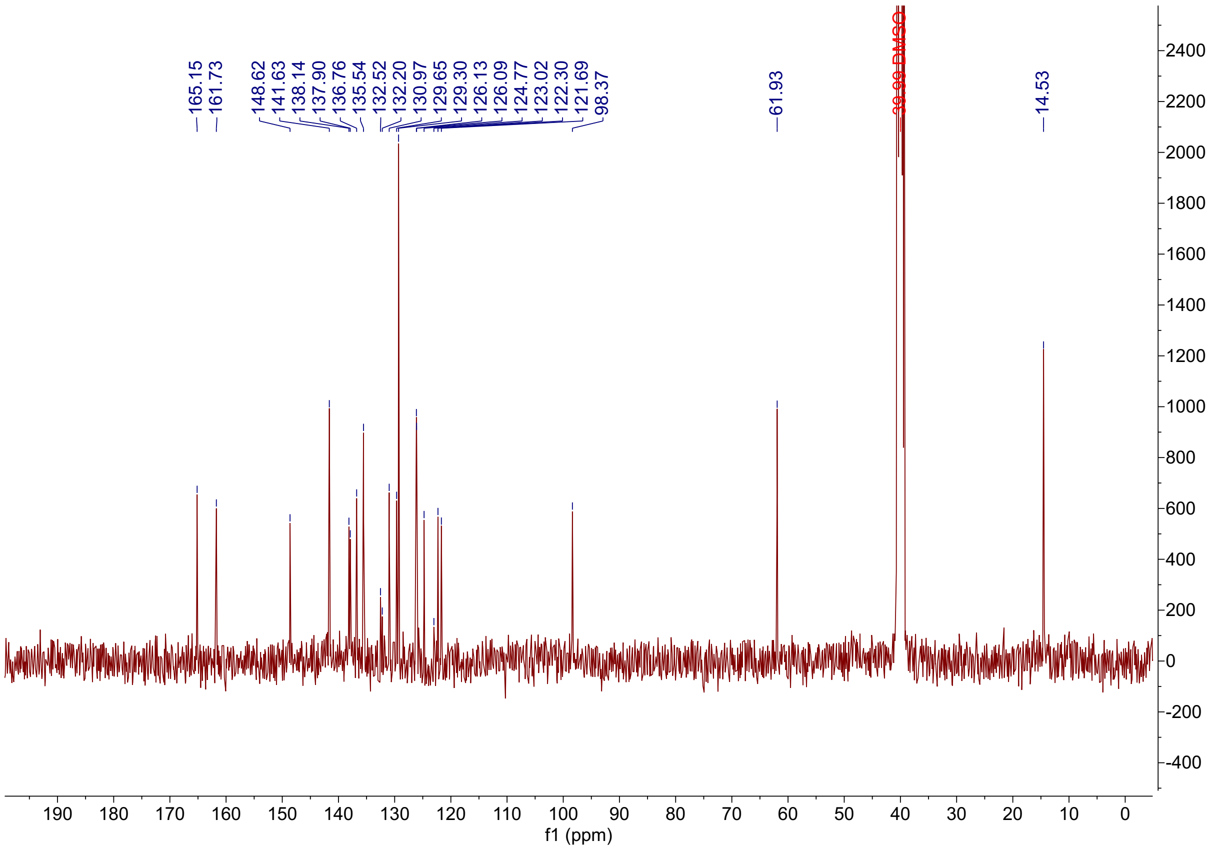

Figure S20. ^{1}HNMR spectrum of compound **2g** (400 MHz, d_6-DMSO)

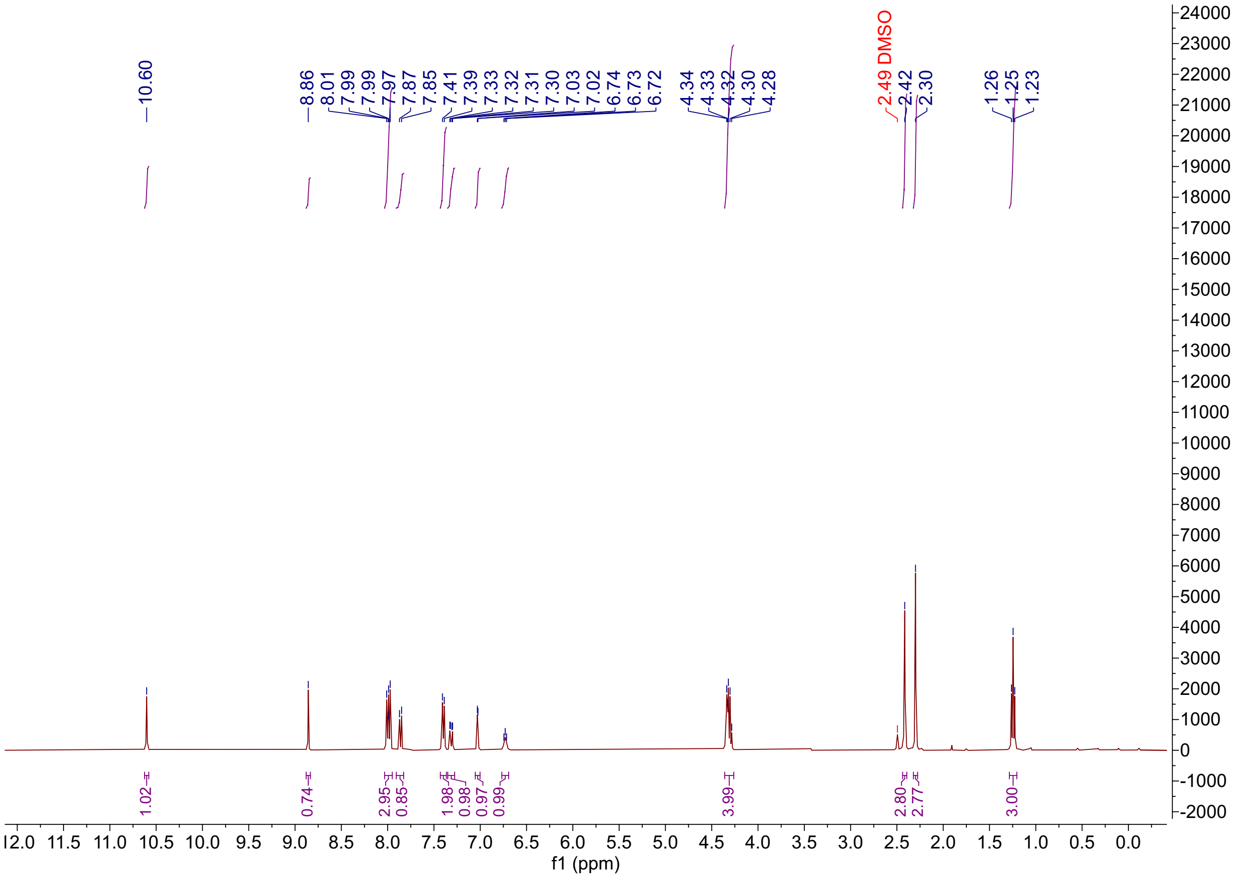

Figure S21. ^{13}CNMR spectrum of compound **2g** (100 MHz, d_6-DMSO)

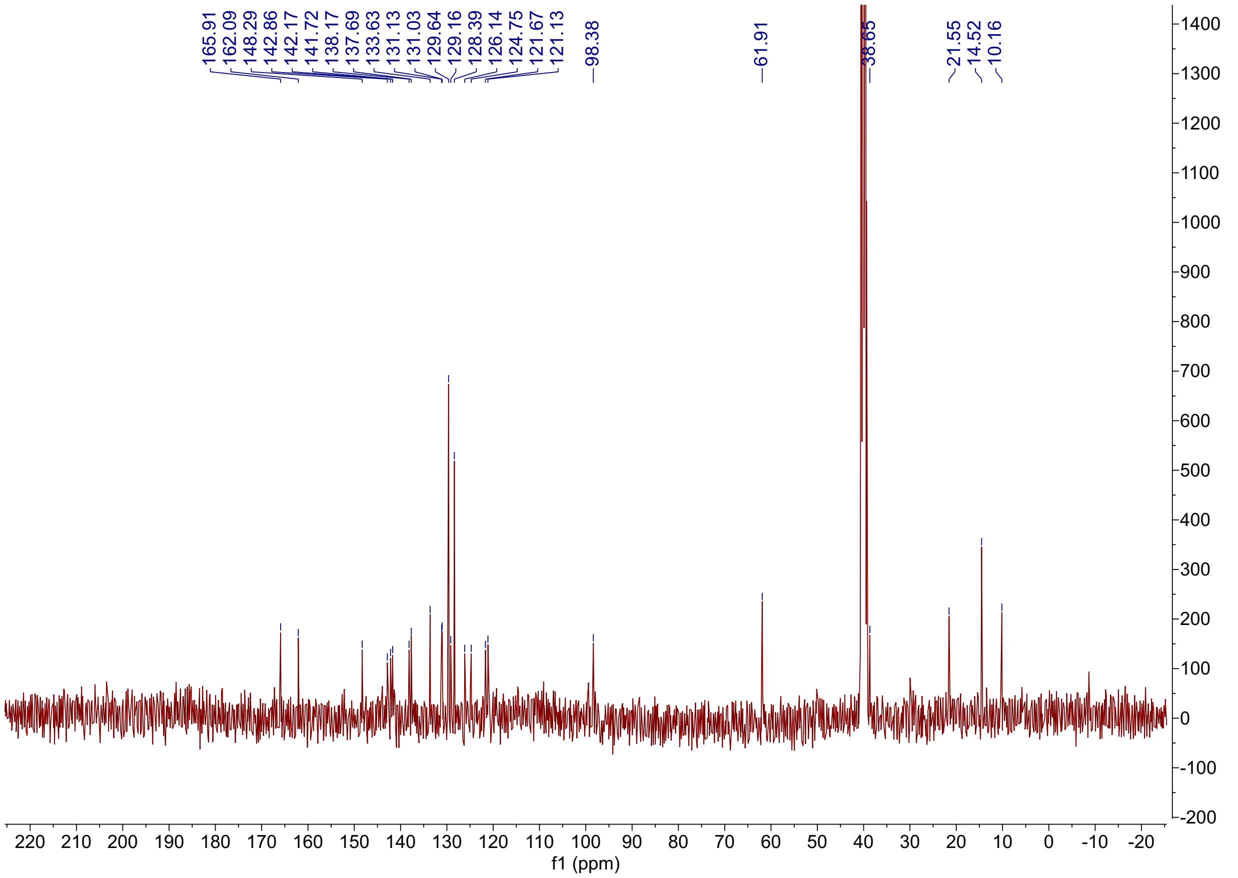

Figure S22. ^{1}HNMR spectrum of compound **2i** (400 MHz, d_6-DMSO)

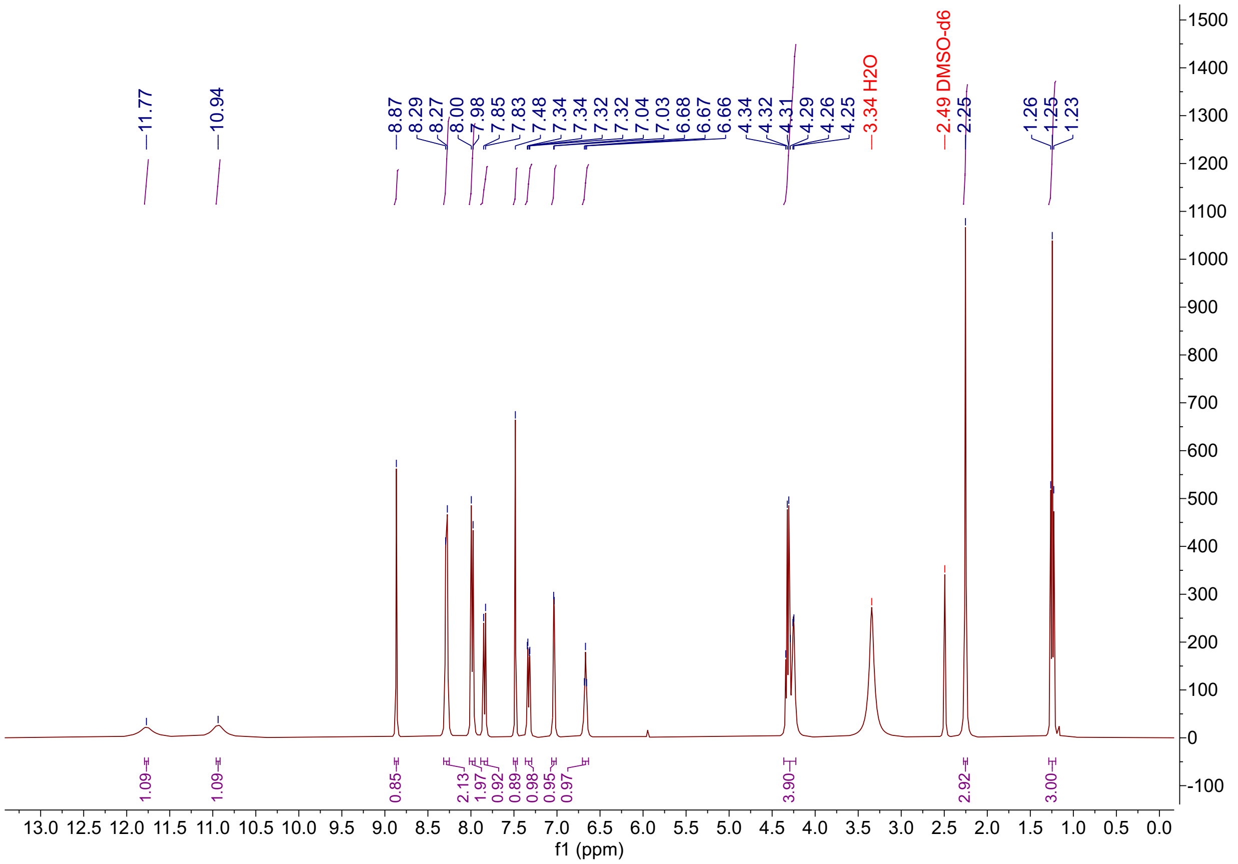

Figure S23. ^{13}CNMR spectrum of compound **2i** (100 MHz, d_6-DMSO)

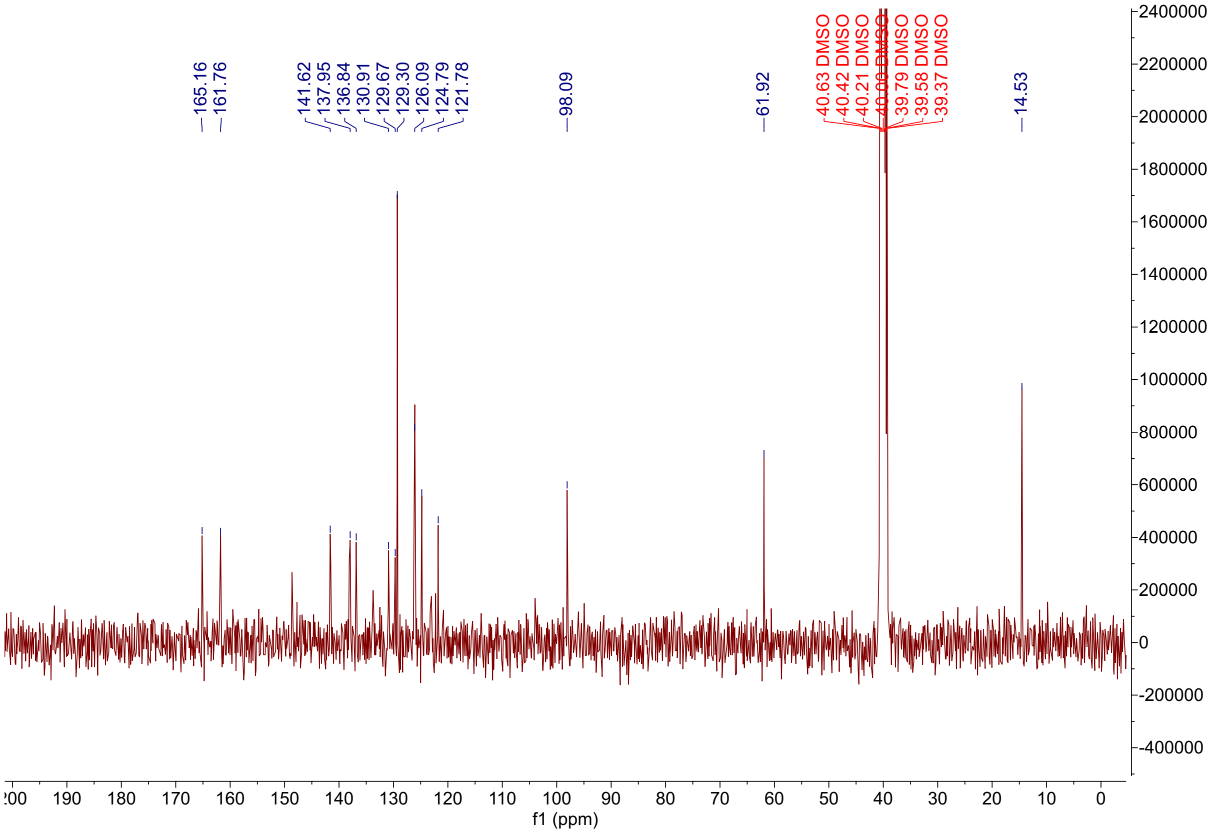

Figures S24. RMSD profiles from independent 200 ns molecular dynamics simulations of the lead protein–ligand complexes.

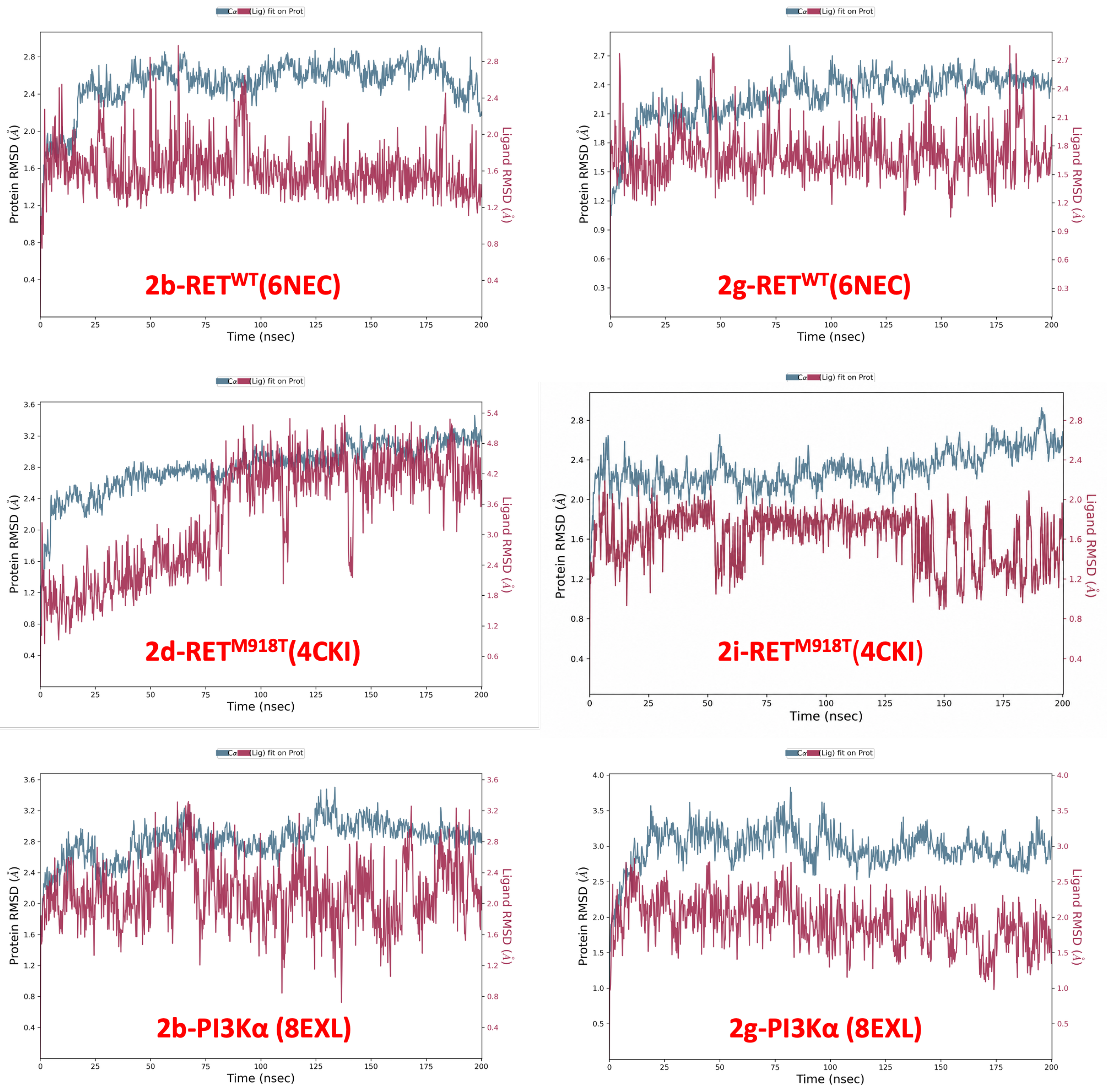

Figure S25. RMSD profiles from independent 50 ns molecular dynamics simulations of the lead protein–ligand complexes.

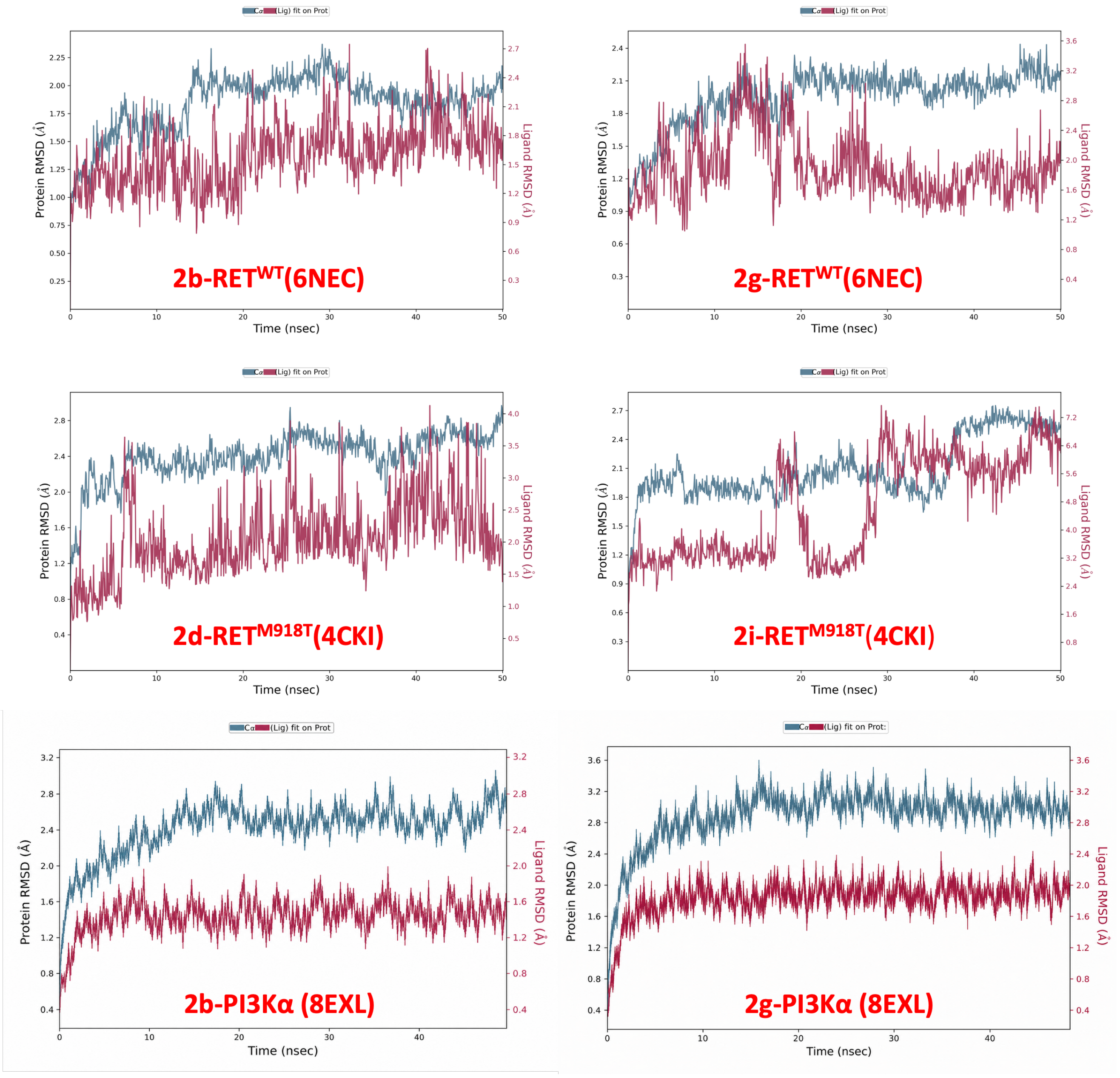

Supporting Tables

Table S1. Prime MM–GBSA energy decomposition of the best ranked ligand–RET complexes obtained from induced fit docking of compounds **2b,g** in the wild–type RET kinase domain (PDB 6NEC). Reported parameters include total Prime Energy and its individual energetic contributions, namely Coulomb, Hbond, Lipo, Packing, Self Contact, Solv GB, and van der Waals (vdW) terms.

Compound	Prime Energy	Prime Coulomb	Prime H bond	Prime Lipo	Prime Packing	Prime Self Cont	Prime Solv GB	Prime vdW
pralsetinib	-12868.5	-8628.42	-132.46	-1706.06	-14.00	-122.34	-2084.77	-1546.97
selpercatinib	-12796.2	-8540.52	-133.36	-1710.39	-13.73	-120.27	-2117.66	-1555.19
Vandetanib	-12788.4	-8526.21	-129.08	-1696.33	-10.52	-121.92	-2129.68	-1550.83
**2b**	-12717.4	-8313.06	-130.37	-1702.33	-12.63	-122.31	-2261.86	-1545.03
**2g**	-12715.6	-8330.35	-134.92	-1700.61	-11.14	-122.04	-2257.80	-1548.39
cabozantinib	-12711.7	-8312.16	-131.68	-1688.52	-14.33	-122.55	-2287.90	-1529.03
**1b**	-12678.1	-8436.64	-133.44	-1681.56	-14.55	-120.24	-2111.39	-1552.09
nintedanib (co-cryst. ligand)	-12641.9	-8362.54	-130.77	-1702.12	-10.18	-120.25	-2169.89	-1534.80

Table S2. Prime MM-GBSA energy decomposition of the best ranked ligand-RET complexes obtained from induced fit docking of compounds **2d,i** in the RET^{M918T} kinase domain (PDB 4CKI). Reported parameters include total Prime Energy and the corresponding Coulomb, Hbond, Lipo, Packing, Self Contact, Solv GB, and van der Waals (vdW) contributions.

Compound	Prime Energy	Prime Coulomb	Prime H bond	Prime Lipo	Prime Packing	Prime Self Cont	Prime Solv GB	Prime vdW
pralsetinib	-13223.7	-8758.98	-141.54	-1726.98	-12.99	-128.04	-2368.54	-1530.05
vandetanib	-13177.7	-8703.32	-139.17	-1724.62	-15.12	-130.67	-2379.72	-1539.42
selpercatinib	-13140.0	-8643.81	-139.93	-1732.13	-13.57	-130.31	-2404.08	-1557.10
**2i**	-13099.5	-8449.26	-141.72	-1730.59	-15.19	-130.42	-2529.72	-1555.90
**2d**	-13078.2	-8430.66	-138.34	-1722.16	-14.85	-130.78	-2534.81	-1551.04
cabozantinib	-13077.2	-8437.01	-139.95	-1722.22	-14.12	-127.94	-2564.97	-1530.95
**1b**	-13057.8	-8561.93	-137.65	-1725.79	-13.59	-130.40	-2373.49	-1552.84
adenosine (co-cryst. ligand)	-13011.7	-8589.65	-134.08	-1674.62	-11.53	-130.77	-2364.55	-1553.27

Table S3. Prime MM-GBSA energy decomposition of the best ranked ligand-PI3Kα complexes obtained from induced fit docking of compounds **2b,g** in the PI3Kα kinase domain (PDB 8EXL). Reported parameters include total Prime Energy and the corresponding Coulomb, Hbond, Lipo, Packing, Self Contact, Solv GB, and van der Waals (vdW) contributions.

Compound	Prime Energy	Prime Coulomb	Prime H bond	Prime Lipo	Prime Packing	Prime Self Cont	Prime Solv GB	Prime vdW
**2b**	-40559.7	-28227.7	-426.8	-5359.7	-57.7	-414.1	-5246.6	-5090.1
omipalisib	-40558.1	-28336.9	-423.5	-5356.4	-48.6	-414.5	-5154.7	-5083.2
**2g**	-40551.5	-28248.1	-423.4	-5355.3	-50.0	-420.6	-5231.4	-5089.6
**1b**	-40512.1	-28315.8	-420.0	-5350.1	-52.9	-415.0	-5117.0	-5089.8
taselisib (co-cryst. ligand)	-40506.2	-28343.9	-425.4	-5364.2	-51.2	-416.6	-5113.4	-5104.5
dactolisib	-40494.5	-28348.5	-417.2	-5369.8	-50.3	-415.1	-5118.6	-5075.7

Bibliography

1. La Monica, G.; Pizzolanti, G.; Baiamonte, C.; Bono, A.; Alamia, F.; Mingoia, F.; Lauria, A.; Martorana, A. Design and Synthesis of Novel Thieno[3,2-c]Quinoline Compounds with Antiproliferative Activity on RET-Dependent Medullary Thyroid Cancer Cells. *ACS Omega* **2023**, *8*, 34640–34649, doi:10.1021/acsomega.3c03578.
2. La Monica, G.; Bono, A.; Alamia, F.; Tocco, D.; Lauria, A.; Martorana, A. Imidazole-Functionalized Thieno[3,2-c]Quinolines as Promising Antiproliferative Agents: Design, Synthesis, NCI-60 Screening, and Computational Analysis. *ACS Omega* **2026**, *11*, 29118–29135, doi:10.1021/acsomega.6c02681.
